# Comparative efficacy of different probiotic strains and preparations for prevention of acute otitis media in children: a systematic review and network meta-analysis

**DOI:** 10.3389/fnut.2026.1847652

**Published:** 2026-07-03

**Authors:** Meiting Ye, Xianjue Xu, Wenrui Huang, Xuelian Du, Yuanxian Liu, Gui Yang

**Affiliations:** 1 Guangzhou University of Chinese Traditional Medicine Shenzhen Clinical College, Shenzhen, Guangdong, China; 2 Shenzhen Traditional Chinese Medicine Hospital, Shenzhen, Guangdong, China; 3 Shenzhen Hospital of Guangzhou University of Chinese Medicine (Futian), Shenzhen, Guangdong, China; 4 Department of Otolaryngology of Longgang Central Hospital and Clinical College Affiliated to Guangzhou University of Chinese Medicine, Shenzhen, China

**Keywords:** acute otitis media, children, network meta-analysis, probiotics, strain-specific

## Abstract

**Background:**

Probiotics are established candidates for preventing acute otitis media (AOM) in children, yet existing meta-analyses treat all probiotic interventions as a single class, precluding strain-specific clinical guidance. This network meta-analysis (NMA) compared the efficacy and safety of individual probiotic strains for pediatric AOM prevention.

**Methods:**

We systematically searched PubMed, Embase, the Cochrane Library, Web of Science, CNKI, WanFang, and CBM from inception to 15 March 2026. Eligible studies were RCTs evaluating probiotic interventions for AOM prevention in children aged 0–18 years. The primary outcome was AOM incidence; secondary outcomes included antibiotic prescription rates, tympanostomy tube placement rates, and adverse events. NMAs were performed using a multivariate random-effects frequentist framework (Stata 17.0). Treatment ranking was estimated using SUCRA values.

**Results:**

Eighteen RCTs (reported in 20 publications) enrolling 4,462 children were included, evaluating seven probiotic nodes (SS-K12, SS-24SMB, BB-12, CBA-L74, LGG, Multi-strain, α-Strep) against placebo. For AOM incidence, only CBA-L74 (OR = 0.27, 95% CI: 0.11–0.68; SUCRA = 89.3%) and SS-K12 (OR = 0.44, 95% CI: 0.20–0.94; SUCRA = 71.8%) achieved statistically significant reductions vs. placebo. Sensitivity analyses excluding two high risk-of-bias trials showed that SS-K12's effect was substantially attenuated and lost significance, whereas CBA-L74 remained robustly effective (OR = 0.28, 95% CI: 0.14–0.56). For antibiotic prescription rates, Multi-strain (OR = 0.45; SUCRA = 87.1%) and LGG (OR = 0.69; SUCRA = 65.3%) demonstrated significant reductions; both were superior to SS-K12 in direct pairwise comparisons. No intervention significantly reduced tympanostomy tube placement rates, though Multi-strain showed the most favorable numerical trend (SUCRA = 93.2%). Among exploratory outcomes, SS-K12 and LGG significantly reduced RTI incidence, while CBA-L74 and LGG significantly reduced AGE incidence, with CBA-L74 ranking first for AGE (SUCRA = 95.9%).

**Conclusions:**

Probiotic efficacy for AOM prevention is highly strain-specific. CBA-L74 demonstrated the most robust and consistent protection across AOM and gastrointestinal outcomes; Multi-strain combinations and LGG were most effective for antibiotic stewardship. These findings provide the first strain-level evidence base to guide rational probiotic selection in pediatric AOM prevention.

**Systematic review registration:**

PROSPERO, https://www.crd.york.ac.uk/prospero/display_record.php?ID=CRD420261360351, identifier CRD420261360351.

## Introduction

Acute otitis media (AOM) is the most common bacterial infection of childhood and the leading indication for pediatric antibiotic prescribing globally. Approximately 316 million incident cases occur annually among children and adolescents, with the highest burden in infants under 1 year of age (1, 2). Beyond acute morbidity, recurrent AOM carries well-established risks of conductive hearing loss, language developmental delay, and surgical intervention; over 413,000 tympanostomy tube placements are performed annually in the United States alone (3). Concurrently, the near-reflexive antibiotic prescribing associated with AOM—immediate antibiotics exceeding 80% of consultations in real-world practice (4)—has accelerated antimicrobial resistance and disrupted the commensal microbiota, reinforcing the urgent need for effective non-antibiotic preventive strategies (5, 6).

Probiotics are biologically plausible candidates for AOM prevention, operating through competitive exclusion of otopathogens from nasopharyngeal epithelium, direct antimicrobial activity via bacteriocins and organic acids, and immunomodulation along the gut–lung axis (7, 8). Two pivotal meta-analyses have provided aggregate support for this approach: the 2019 Cochrane review by Scott et al. (3) (17 RCTs, *n* = 3,488) reported a significant reduction in AOM incidence (RR 0.77, 95% CI 0.63–0.93; NNTB = 10), and Mosquera et al. (9) corroborated an approximately 20% relative risk reduction across 16 RCTs. Both reviews also demonstrated meaningful reductions in antibiotic use and acceptable safety profiles.

However, these meta-analyses share a critical methodological limitation: all probiotic interventions are collapsed into a single node in pairwise comparison against placebo, irrespective of profound inter-strain biological heterogeneity. Probiotic efficacy is strain-specific, not a class effect (10). *Streptococcus salivarius* K12 acts through targeted bacteriocin production at the oropharyngeal mucosa (11); Lactobacillus rhamnosus GG relies on mucosal adhesion pili and immunomodulatory effector proteins (7); *Bifidobacterium animalis* subsp. *lactis* BB-12 operates primarily via systemic gut–lung axis immunostimulation (12). The extreme heterogeneity reported in existing meta-analyses (I^2^ = 72–100%) directly reflects this unresolved strain-level variance, rendering pooled estimates clinically unactionable for strain selection.

Network meta-analysis (NMA) provides the methodological framework to address this gap. By integrating direct and indirect comparative evidence within a unified statistical network, NMA permits simultaneous estimation of relative efficacy across multiple interventions that have never been evaluated in head-to-head trials—a near-universal condition in the probiotic AOM literature (13). SUCRA values and *P*-scores derived from the network further enable probabilistic ranking of competing strains, translating complex multi-arm evidence into clinically interpretable hierarchies (14, 15).

This systematic review and NMA aims to compare the efficacy and safety of specific probiotic strains and preparations for AOM prevention in children. The primary outcome is AOM incidence; secondary outcomes include antibiotic use, tympanostomy tube placement rates, and adverse events. To our knowledge, this is the first NMA to evaluate probiotic interventions for pediatric AOM prevention at the strain level, and is intended to provide actionable guidance for clinical decision-making in this high-burden condition.

## Methods

This systematic review and network meta-analysis was conducted in accordance with the PRISMA 2020 statement and its extension for Network Meta-Analyses (PRISMA-NMA). The study protocol was prospectively registered in the PROSPERO international database (registration number: CRD420261360351) to ensure methodological transparency and minimize the risk of selective reporting.

### Search strategy

We systematically searched PubMed, Embase, Web of Science, the Cochrane Central Register of Controlled Trials (CENTRAL), CNKI, WanFang, and CBM from database inception to 15 March 2026. Two reviewers independently conducted searches and screened records against predefined eligibility criteria; discrepancies were resolved by discussion or adjudication by a third reviewer. In addition, ClinicalTrials.gov and the WHO International Clinical Trials Registry Platform (ICTRP) were searched for ongoing or unpublished trials, and reference lists of included studies and relevant systematic reviews were manually screened to identify additional eligible records. The complete search strategies for each database are provided in Supplementary Appendix 1.

### Eligibility criteria

Eligible studies were RCTs evaluating probiotic interventions for the prevention of AOM in children aged 0 to 18 years. Interventions included any probiotic preparation—administered as a single strain or multi-strain combination, in any formulation (capsule, powder, liquid, and nasal spray) and by any route—with or without concurrent standard care. Trials in which probiotics were used as adjuncts to standard prophylaxis were also eligible. Heat-inactivated probiotic-derived preparations [i.e., postbiotics, such as the heat-killed *Lactobacillus paracasei* CBA-L74 used in the Nocerino et al. (16) and Corsello et al. (17) trials] were also eligible on the basis of their mechanistic relevance to AOM prevention and their increasing recognition in current ISAPP consensus statements; throughout this manuscript we explicitly label CBA-L74 as a postbiotic, and use the term “probiotic interventions” to denote the combined probiotic-plus-postbiotic comparator set for ease of presentation. Eligible comparators were placebo, no treatment, or an alternative probiotic preparation. Studies restricted to prebiotic-only interventions, or enrolling participants with immunodeficiency, primary ciliary dyskinesia, craniofacial anomalies, or other conditions predisposing to secondary AOM, were excluded.

The primary outcome was the incidence of AOM episodes during the intervention or follow-up period. Secondary outcomes included antibiotic prescription rates, tympanostomy tube placement rates, and the incidence of adverse events. Studies not reporting at least the primary outcome were excluded. Only peer-reviewed, full-text articles published in English or Chinese were included; conference abstracts, non-randomized studies, and crossover trials were excluded.

### Screening process

Records retrieved from all databases were imported into EndNote 20.4.1 (Clarivate Analytics, Philadelphia, PA, USA), and duplicates removed. Screening proceeded in two stages: independent title-and-abstract screening followed by full-text review of potentially eligible records, with disagreements at each stage resolved by discussion or consultation with a third reviewer.

### Data extraction

Predesigned extraction forms were developed *a priori*. Two reviewers independently extracted data; discrepancies were resolved by consensus. Extracted information included study characteristics (year, country, design, and sample size), participant details (age, setting, and AOM risk profile), intervention features (probiotic genus, species, strain designation, CFU dose, formulation, administration route, and treatment duration), comparator characteristics, and outcome data (AOM incidence, antibiotic use, tympanostomy rate, and adverse events) at all reported timepoints.

### Quality assessment

Methodological quality of included RCTs was assessed using the Cochrane Risk of Bias 2.0 (RoB 2) tool, examining random sequence generation, allocation concealment, blinding of participants and outcome assessors, completeness of outcome data, and selective reporting. Two reviewers independently performed assessments, with discrepancies resolved by consensus. The certainty of evidence for each outcome was graded using the CINeMA (Confidence in Network Meta-Analysis) framework across six domains: within-study bias, reporting bias, indirectness, imprecision, heterogeneity, and inconsistency. Intransitivity was evaluated by comparing key effect modifiers—including participant age, clinical setting (day-care vs. community), baseline AOM risk, and probiotic administration route—between studies contributing direct and indirect evidence.

### Statistical analysis

NMAs were performed using Stata 17.0 with the mvmeta command under a multivariate random-effects frequentist framework. The primary outcome (AOM incidence) and binary secondary outcomes (antibiotic use, tympanostomy placement) were analyzed as risk ratios (RRs) with 95% confidence intervals (CIs). Between-study heterogeneity was estimated using restricted maximum likelihood (REML), with a common heterogeneity variance (τ^2^) assumed across treatment contrasts and a between-study correlation fixed at 0.5. The transitivity assumption—namely, that the distribution of effect modifiers is broadly comparable across studies contributing direct and indirect evidence—was qualitatively evaluated by tabulating participant age, day-care vs. community setting, baseline AOM risk, probiotic daily dose (CFU), formulation vehicle, and administration route across all studies in each network (Supplementary Appendix 7). Potential violations of transitivity are interpreted explicitly in the Limitations section.

## Results

### Literature selection

**Figure 1:**
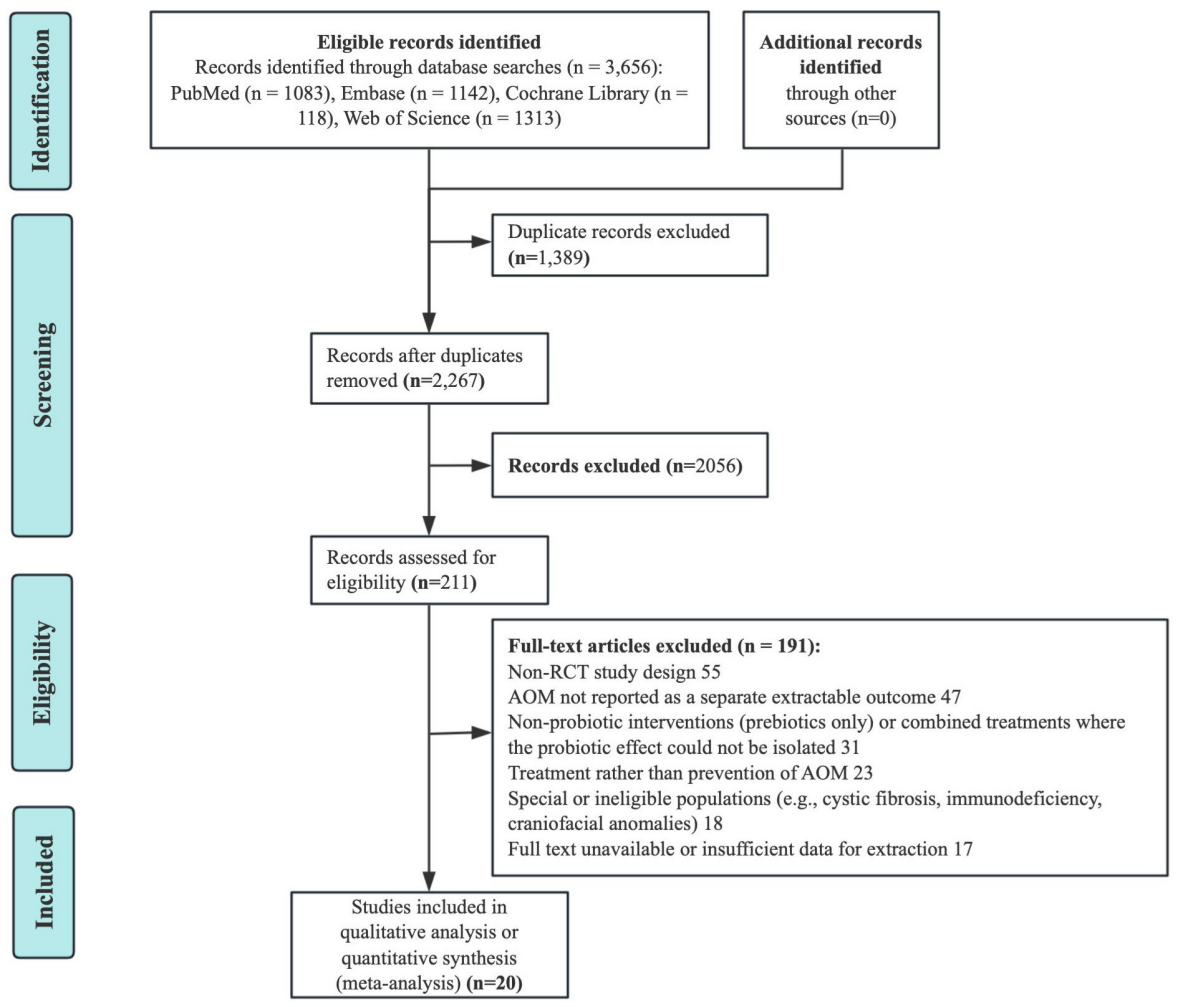
PRISMA flow diagram of study identification and selection process.

A total of 3,656 records were retrieved from four databases: PubMed (*n* = 1,083), Embase (*n* = 1,142), the Cochrane Library (*n* = 118), and Web of Science (*n* = 1,313). After removing 1,389 duplicates, 2,267 records remained for title and abstract screening, of which 2,056 were excluded as irrelevant. The remaining 211 full-text articles were assessed for eligibility; 191 were subsequently excluded due to non-RCT study design (*n* = 55), AOM not reported as a separate extractable outcome (*n* = 47), non-probiotic or combined interventions where the probiotic effect could not be isolated (*n* = 31), treatment rather than prevention of AOM (*n* = 23), ineligible populations (*n* = 18), or unavailable or insufficient full-text data (*n* = 17). Ultimately, 18 RCTs reported in 20 publications (16–35) met the inclusion criteria and were included in the qualitative and quantitative synthesis; Taipale et al. (29, 30) and Maldonado et al. (25)/Maldonado-Lobón et al. (26) each represent two reports from a single trial cohort and were treated as one study throughout all analyses (Figure 1).

### Study characteristics

The 18 included RCTs (reported in 20 publications, published between 2001 and 2024) enrolled a total of 4,462 participants across 9 countries, with sample sizes ranging from 36 [Tano et al. (20)] to 827 [Sarlin et al. (34)]. Participants were children aged 1 month to 13 years; the majority were preschool-aged children attending day care centers or kindergartens. Intervention duration ranged from 30 days [Karpova et al. (33)] to 23 months [Taipale et al. (30)], with follow-up periods extending up to 21 months [Stecksén-Blicks et al. (22)]. Sixteen trials adopted a double-blind placebo-controlled design; two used open-label untreated controls [Di Pierro et al. (31); Karpova et al. (33)]. Seven probiotic nodes were identified: SS-K12 (3 RCTs; *n* = 1,268), SS-24SMB (1 RCT; *n* = 97), BB-12 (2 RCTs; *n* = 319), CBA-L74 (2 RCTs; *n* = 434), LGG (3 RCTs; n = 1,100), multi-strain combinations (3 RCTs; *n* = 614), and α-Strep (2 RCTs; *n* = 144). Fifteen trials administered interventions orally; three used intranasal delivery [Marchisio et al. (28); Roos et al. (19); Tano et al. (20)]. Daily doses ranged from 1–2 × 10^8^ to 4.1 × 10^10^ CFU (See Table 1).

**Table 1:** Baseline characteristics of included.

Study	Country	Design	Age	Total *N* (trt/ctrl)	Probiotic (Strain)	Route	Daily dose	Duration	Control	Follow-up
Hatakka et al. (18)	Finland	DB-RCT	1–6 yr	571 (282/289)	LGG	Oral	1–2 × 108 CFU	7 mo	Placebo	7 mo
Roos et al. (19)	Sweden	DB-RCT	6 mo−6 yr	108 (53/55)	α-haemolytic streptococci	Intranasal	>5 × 106 CFU/ml	2 × 10-day courses	Placebo	3 mo
Tano et al. (20)	Sweden	DB-RCT	≤ 3 yr	36 (16/20)	α-haemolytic streptococci	Intranasal	>10^6^ CFU/spray	4 mo	Placebo	6 mo
Hatakka et al. (21)	Finland	DB-RCT	10 mo−6 yr	309 (155/154)	LGG + LC705 + B. breve 99 + P. freudenreichii JS	Oral	8–9 × 10^9^ CFU/strain	6 mo	Placebo	6 mo
Stecksén-Blicks et al. (22)	Sweden	Cluster-RCT	1–5 yr	248 (133/115)	L. rhamnosus LB21	Oral	1.5 × 10^9^ CFU	21 mo	Placebo	21 mo
Rautava et al. (23)	Finland	DB-RCT	< 2–12 mo	81 (38/43)	LGG + BB-12	Oral	2 × 10^10^ CFU	~10 mo	Placebo	To age 12 mo
Hojsak et al. (24)	Croatia	DB-RCT	13–86 mo	281 (139/142)	LGG	Oral	10^9^ CFU	3 mo	Placebo	3 mo
Maldonado et al. (25, 26)	Spain	DB-RCT	6–12 mo	215 (117/98)	L. fermentum CECT5716	Oral	2 × 10^8^ CFU	6 mo	Placebo	6 mo
Cohen et al. (27)	France	DB-RCT	7–13 mo	224 (112/112)	S. thermophilus NCC2496 + S. salivarius DSM13084 + L. rhamnosus LPR	Oral	107 CFU/g	12 mo	Placebo	12 mo
Marchisio et al. (28)	Italy	DB-RCT	1–5 yr	97 (50/47)	SS-24SMB	Intranasal	2 × 10^10^ CFU	3 mo	Placebo	6 mo
Nocerino et al. (16)	Italy	DB-RCT	12–48 mo	288 (141+5/127)^†^	L. paracasei CBA L74 (inactivated)	Oral	~4.1 × 10^10^ CFU	3 mo	Placebo	3 mo
Taipale et al. (29, 30)	Finland	DB-RCT	1 mo−2 yr	109 (55/54)	BB-12	Oral	10^10^ CFU	~23 mo	Placebo	To age 2 yr
Di Pierro et al. (31)	Italy	Open-label RCT	33–45 mo	222 (111/111)	SS-K12	Oral	≥10^9^ CFU	6 mo	Placebo	9 mo
Hojsak et al. (32)	Croatia	DB-RCT	1.4–7.5 yr	210 (104/106)	BB-12	Oral	10^9^ CFU	3 mo	Placebo	3 mo
Karpova et al. (33)	Russia	Open-label RCT	6–7 yr	219 (113/106)	SS-K12	Oral	NR	30 days	Placebo	3 mo
Corsello et al. (17)	Italy	MC DB-RCT	12–48 mo	146 (73/73)	L. paracasei CBA L74 (inactivated)	Oral	NR	3 mo	Placebo	3 mo
Sarlin et al. (34)	Finland	DB-RCT	1–6 yr	827 (413/414)	SS-K12	Oral	10^9^ CFU	6 mo	Placebo	6 mo
Paduchová et al. (35)	Slovakia	DB-RCT	3–10 yr	127 (65/62)	CUL21 + CUL60 + CUL20 + CUL34	Oral	1.25 × 10^10^ CFU	6 mo	Placebo	6 mo

^†^Two intervention arms (fermented milk *n* = 141; fermented rice *n* = 123) vs. a shared placebo group (*n* = 127); both arms assigned to CBA-L74. mo: months; yr: years; NR: not reported.

### Risk of bias and consistency

**Figure 2:**
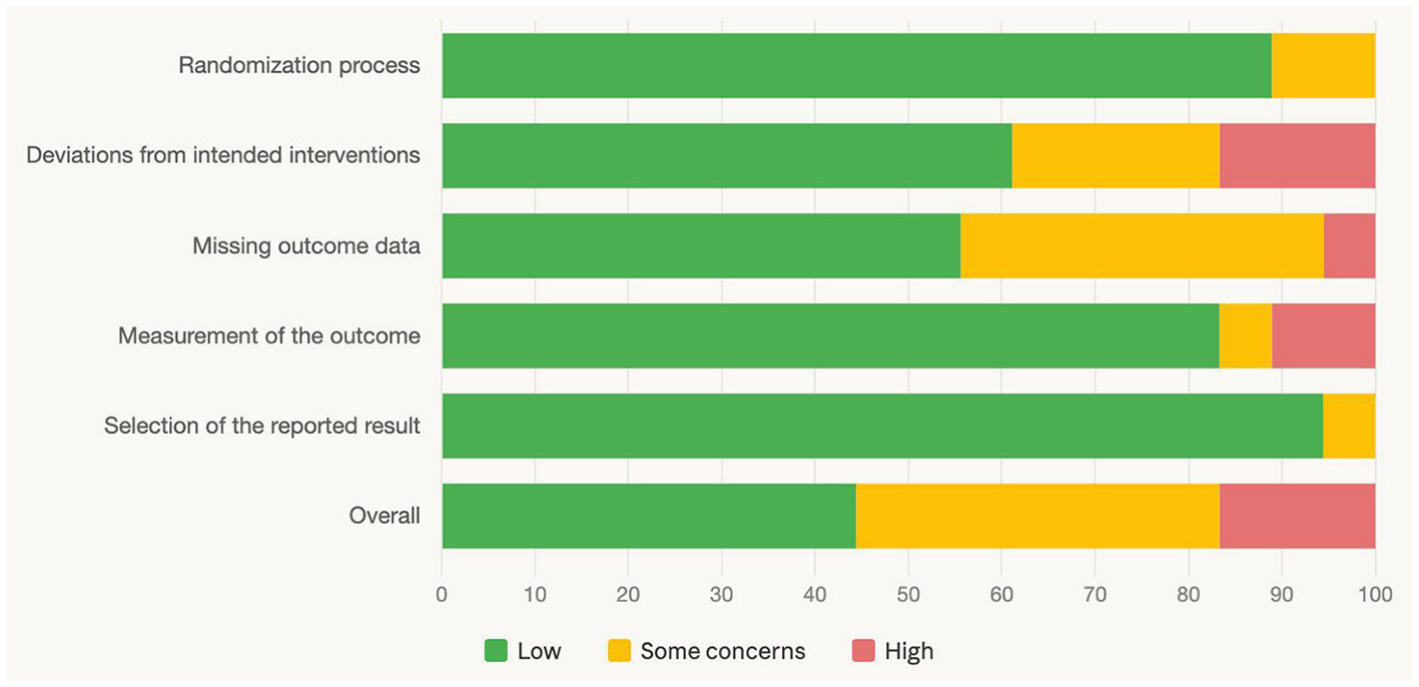
Risk of bias assessment of included studies.

Risk of bias was assessed across all 18 included RCTs using the Cochrane RoB 2 tool. Eight studies were rated as low overall risk of bias [Hatakka et al. (18, 21); Hojsak et al. (24); Cohen et al. (27); Nocerino et al. (16); Hojsak et al. (32); Corsello et al. (17); Sarlin et al. (34)]. Seven studies were rated as raising some concerns [Roos et al. (19); Tano et al. (20); Stecksén-Blicks et al. (22); Rautava et al. (23); Maldonado et al. (25); Marchisio et al. (28); Paduchová et al. (35)], primarily due to incomplete blinding of participants or outcome assessors, substantial missing outcome data without adequate imputation, or uncertainty regarding adherence to the intended intervention. Three studies were rated as high overall risk of bias [Taipale et al. (29, 30); Di Pierro et al. (31); Karpova et al. (33)]. For Taipale et al. (29)/Taipale et al. (30), concerns arose from serious deviations from the intended intervention and high attrition over the extended follow-up period. Di Pierro et al. (31) and Karpova et al. (33) were open-label trials with untreated controls, introducing high risk in the domains of deviations from intended interventions and measurement of the outcome. These two studies will be excluded in sensitivity analyses to assess the robustness of the primary findings (Figure 2 & Supplementary Appendix 2).

For the consistency assessment, no significant inconsistency was detected between direct and indirect comparisons across the network. The estimated τ^2^ values indicated low to moderate heterogeneity within the network, suggesting overall robust model consistency (Supplementary Appendix 3). Additionally, funnel plots showed no evident asymmetry, suggesting a low likelihood of small-study or publication bias (Supplementary Appendix 4).

### Incidence of AOM episodes

**Figure 3:**
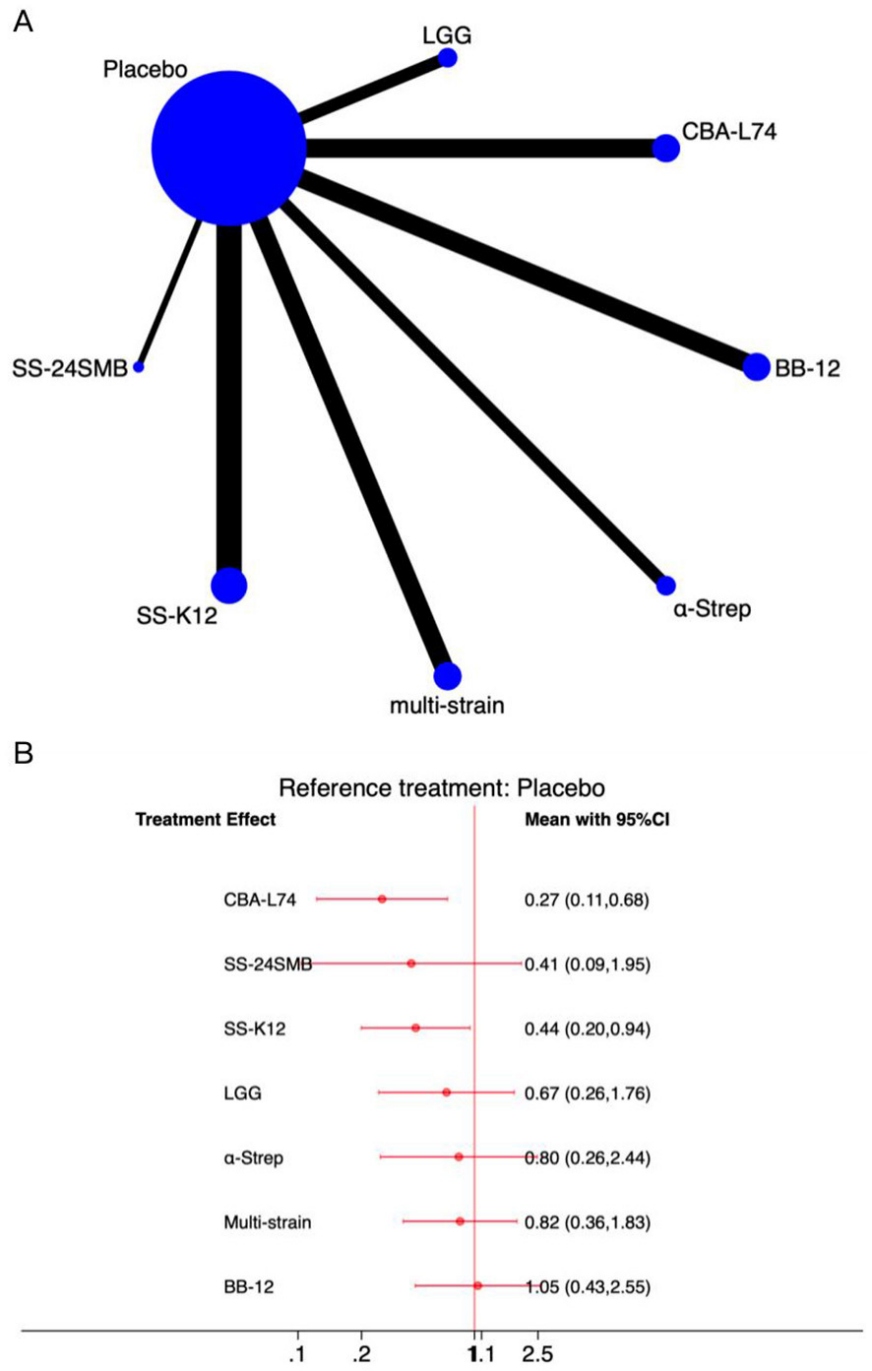
Network and forest plot of Incidence of AOM episodes.

For the primary outcome of AOM incidence, the network meta-analysis incorporated 18 studies enrolling 3,913 participants across 7 intervention nodes (SS-K12, SS-24SMB, BB-12, CBA-L74, LGG, Multi-strain, and α-Strep) plus the reference node Placebo (Figure 3A). Placebo served as the central hub of the network, with all evidence derived from direct comparisons against Placebo; no head-to-head comparisons between active interventions were identified, and the network adopted a star-shaped topology without closed loops. Compared with Placebo, two interventions achieved statistically significant reductions in AOM incidence (Figure 3B). CBA-L74 demonstrated the greatest relative risk reduction (OR = 0.27, 95% CI: 0.11–0.68, SUCRA = 89.3%), followed by SS-K12 (OR = 0.44, 95% CI: 0.20–0.94, SUCRA = 71.8%) (Supplementary Appendix 5, Figure 5.1). In pairwise comparisons from the league table, only the contrast between BB-12 and CBA-L74 was statistically significant (OR = 3.89, 95% CI: 1.08–14.04) (Supplementary Appendix 6, Table 6.1). No other between-intervention differences reached statistical significance.

### Antibiotic prescription rates

**Figure 4:**
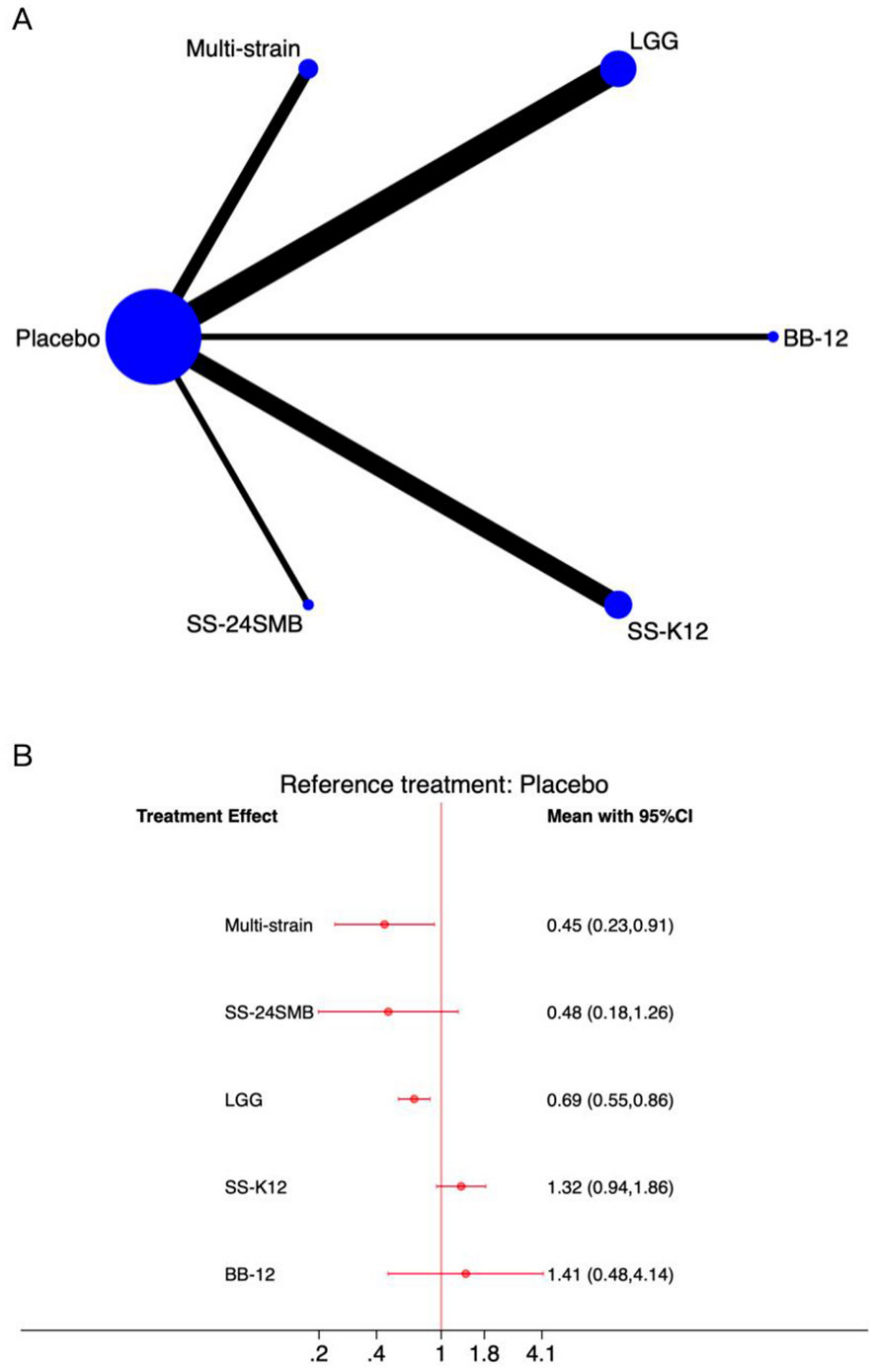
Network and forest plot of Antibiotic prescription rates.

For the secondary outcome of antibiotic prescription rates, the network incorporated 5 intervention nodes (SS-K12, SS-24SMB, BB-12, LGG, and Multi-strain) plus Placebo, adopting a star-shaped topology with Placebo as the central hub (Figure 4A). LGG and SS-K12 were the most densely connected nodes; no closed loops were identified. Compared with Placebo, two interventions achieved statistically significant reductions in antibiotic prescription rates (Figure 4B). Multi-strain demonstrated the greatest effect (OR = 0.45, 95% CI: 0.23–0.91, SUCRA = 87.1%), followed by LGG (OR = 0.69, 95% CI: 0.55–0.86, SUCRA = 65.3%) (Supplementary Appendix 5, Figure 5.2). In pairwise comparisons from the league table, both LGG and Multi-strain were associated with significantly lower antibiotic prescription rates relative to SS-K12 (OR = 0.52, 95% CI: 0.35–0.78; and OR = 0.34, 95% CI: 0.16–0.75, respectively) (Supplementary Appendix 6, Table 6.2). No other between-intervention differences reached statistical significance.

### Tympanostomy tube placement rates

**Figure 5:**
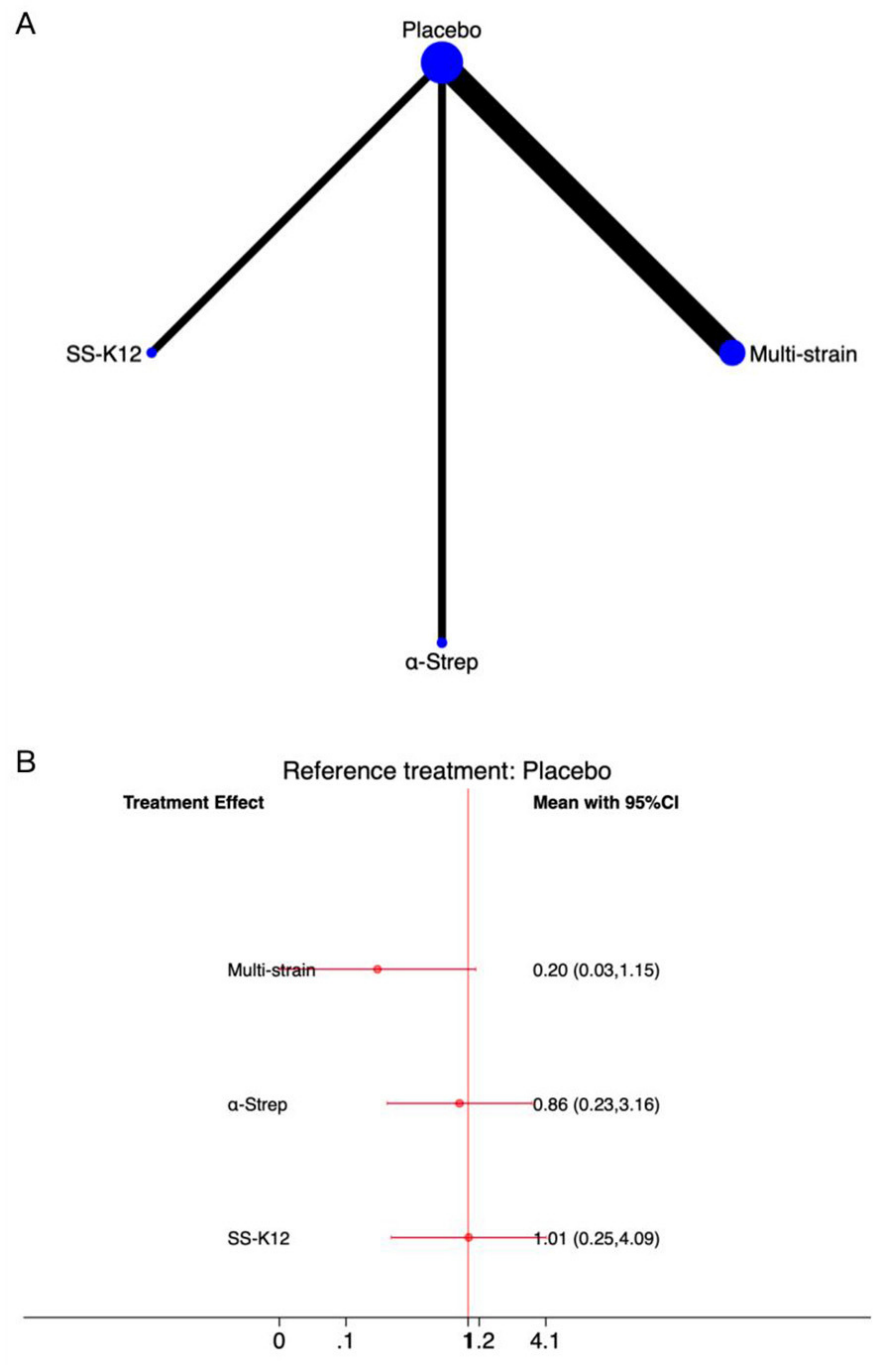
Network and forest plot of Tympanostomy tube placement rates.

For the secondary outcome of tympanostomy tube placement rates, the network incorporated 3 intervention nodes (SS-K12, Multi-strain, and α-Strep) plus Placebo (Figure 5A). The network adopted a star-shaped topology with Placebo as the central hub; no closed loops were identified. No intervention achieved a statistically significant reduction in tympanostomy tube placement rates relative to Placebo (Figure 5B). Although Multi-strain showed a numerically lower point estimate (OR = 0.20, 95% CI: 0.03–1.15), the wide confidence interval crossing the null precluded definitive conclusions. SUCRA-based ranking suggested a potential ordering of Multi-strain (93.2%), α-Strep (42.3%), SS-K12 (32.4%), and Placebo (32.1%) (Supplementary Appendix 5, Figure 5.3); however, given the absence of statistical significance across all comparisons and the limited number of contributing studies, these rankings should be interpreted with caution. No statistically significant differences were identified in pairwise comparisons from the league table (Supplementary Appendix 6, Table 6.3).

### Safety outcome

**Figure 6:**
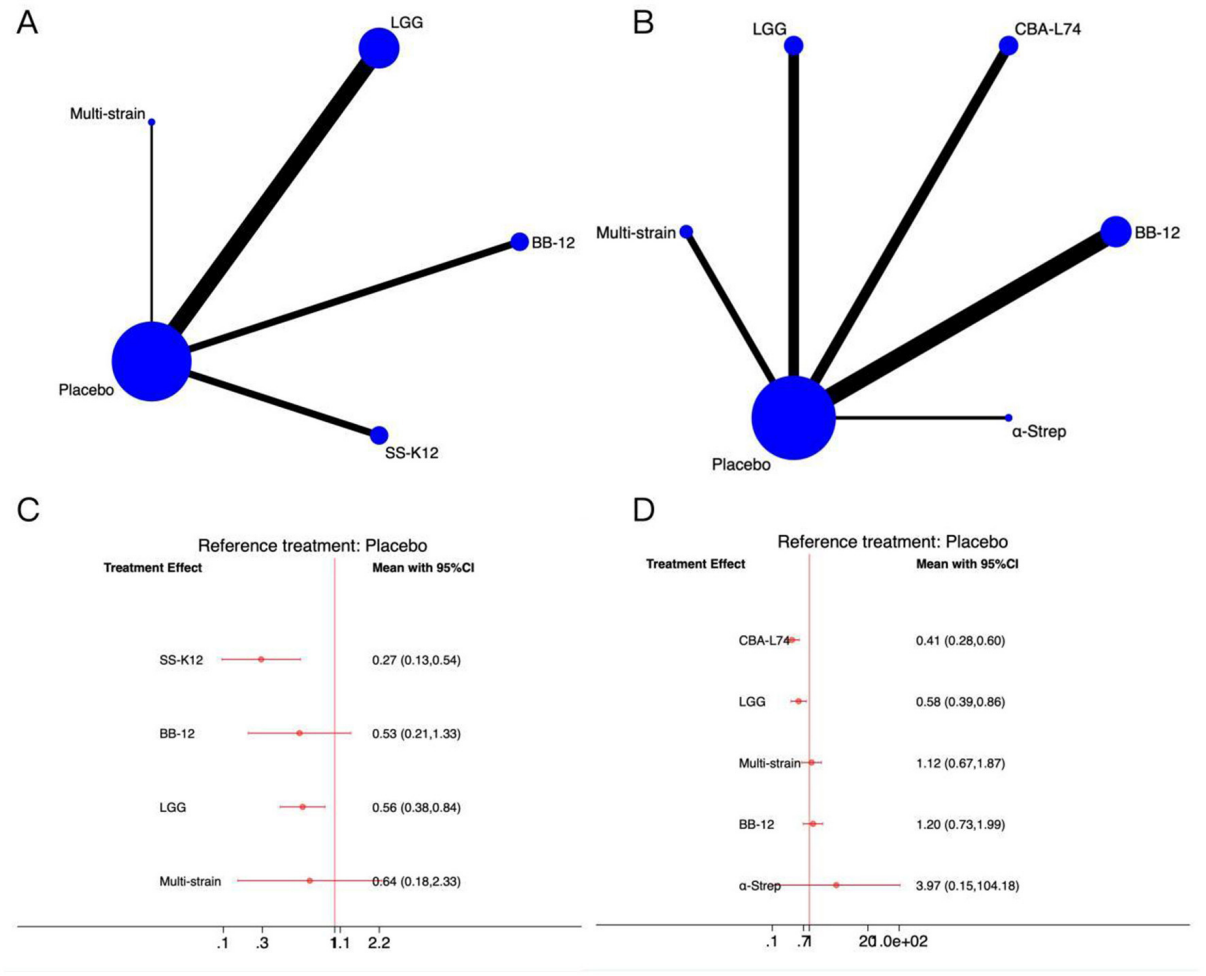
Network and forest plot of RTI and AGE.

For the exploratory outcome of RTI incidence, the network incorporated 4 intervention nodes (SS-K12, BB-12, LGG, and Multi-strain) plus Placebo, with Placebo as the central hub in a star-shaped topology (Figure 6A). Compared with Placebo, two interventions achieved statistically significant reductions in RTI incidence (Figure 6C): SS-K12 (OR = 0.27, 95% CI: 0.13–0.54, SUCRA = 93.0%) and LGG (OR = 0.56, 95% CI: 0.38–0.84, SUCRA = 51.5%) (Supplementary Appendix 5, Figure 5.4). No statistically significant differences were identified in pairwise comparisons from the league table (Supplementary Appendix 6, Table 6.4).

For the exploratory outcome of AGE incidence, the network incorporated 5 intervention nodes (BB-12, CBA-L74, LGG, Multi-strain, and α-Strep) plus Placebo (Figure 6B). Compared with Placebo, two interventions achieved statistically significant reductions in AGE incidence (Figure 6D): CBA-L74 (OR = 0.41, 95% CI: 0.28–0.60, SUCRA = 95.9%) and LGG (OR = 0.58, 95% CI: 0.38–0.88, SUCRA = 78.8%) (Supplementary Appendix 5, Figure 5.5). In pairwise comparisons from the league table, BB-12 was associated with significantly higher AGE incidence relative to both CBA-L74 (OR = 2.93, 95% CI: 1.56–5.51) and LGG (OR = 2.09, 95% CI: 1.10–3.96). Additionally, both CBA-L74 and LGG demonstrated significantly lower AGE incidence compared with Multi-strain (OR = 0.37, 95% CI: 0.19–0.70; and OR = 0.52, 95% CI: 0.27–0.99, respectively). No other between-intervention differences reached statistical significance (Supplementary Appendix 6, Table 6.5).

### Certainty of evidence (CINeMA)

Confidence in the network estimates was assessed using the CINeMA framework across six domains (within-study bias, reporting bias, indirectness, imprecision, heterogeneity, and inconsistency). For the primary outcome of AOM incidence, the certainty of the placebo-controlled estimate was rated moderate for CBA-L74, low for SS-K12 (downgraded for within-study bias driven by the two open-label trials and further attenuated in sensitivity analysis), and very low for SS-24SMB and α-Strep (downgraded for imprecision and very sparse direct evidence). For antibiotic prescription, certainty was rated moderate for LGG and low for Multi-strain (downgraded for imprecision and heterogeneity in antibiotic-indication definitions). For tympanostomy tube placement, certainty was very low across all contrasts owing to imprecision and few events. All between-strain (active vs. active) contrasts carried at least one downgrade for indirectness because they relied exclusively on indirect evidence within the star-shaped network. Full CINeMA judgements are reported in Supplementary Appendix 9.

### Sensitivity analysis

To assess the robustness of the primary outcome results, a pre-specified sensitivity analysis was conducted by excluding the two studies rated as high overall risk of bias—Di Pierro et al. (31) and Karpova et al. (33)—both of which were open-label trials with untreated controls that contributed exclusively to the SS-K12 node. The results were substantially altered for SS-K12 but remained stable for all other interventions. CBA-L74 retained its statistically significant superiority over placebo with a marginally tightened confidence interval (OR = 0.28, 95% CI: 0.14–0.56), confirming the robustness of its primary outcome estimate (Supplementary Appendix 8). In contrast, SS-K12 lost statistical significance entirely upon exclusion of the two high-risk studies, with its point estimate shifting markedly from 0.44 to 1.46 (95% CI: 0.68–3.14) and its ranking falling from second to last among active interventions. The estimates for all remaining nodes—SS-24SMB, LGG, α-Strep, Multi-strain, and BB-12—remained directionally consistent with the primary analysis, with no intervention other than CBA-L74 achieving statistical significance.

## Discussion

### Primary findings

This network meta-analysis synthesized evidence from 18 RCTs (reported in 20 publications) enrolling 4,462 children to compare the strain-level efficacy of probiotic interventions for the prevention of AOM and related outcomes. To our knowledge, this represents the first NMA to evaluate probiotic strains at the individual strain level for pediatric AOM prevention. Several key findings emerged. For the primary outcome of AOM incidence, CBA-L74 and SS-K12 were the only interventions to achieve statistically significant reductions relative to placebo, with CBA-L74 ranking highest by SUCRA (89.3%) and SS-K12 second (71.8%). For antibiotic prescription rates, Multi-strain combinations and LGG demonstrated significant reductions vs. placebo, and both were superior to SS-K12 in direct pairwise comparisons. For tympanostomy tube placement, no intervention reached statistical significance, though Multi-strain showed the most favorable numerical trend. Among exploratory outcomes, SS-K12 and LGG significantly reduced RTI incidence, while CBA-L74 and LGG significantly reduced AGE incidence—with CBA-L74 consistently ranking first across both infectious outcomes. Collectively, these findings challenge the prevailing assumption that probiotic effects are a class-wide phenomenon, demonstrating instead that protective efficacy is highly strain-specific and outcome-dependent.

### AOM primary outcome

The superior performance of CBA-L74 in preventing AOM is particularly noteworthy given that it is a heat-inactivated preparation—a postbiotic rather than a live probiotic—processed at 85 °C for 20 s (36). This finding fundamentally challenges the premise that mucosal colonization by viable microorganisms is a prerequisite for clinical benefit (36). The protective mechanism of CBA-L74 operates through the gut–lung axis: its fermentation metabolites and cell wall components—including peptidoglycans, lipoteichoic acid, and CpG-rich DNA—act as pathogen-associated molecular patterns (PAMPs) that engage Toll-like receptors in gut-associated lymphoid tissue, triggering the release of antimicrobial peptides (α-defensin HNP1-3, β-defensin-2, cathelicidin LL-37) and stimulating mucosal IgA production (37, 38). Simultaneously, CBA-L74 promotes the expansion of butyrate-producing taxa, generating short-chain fatty acids that reinforce tight junction proteins (Occludin, Zonula occludens-1) and drive regulatory T-cell differentiation (39). The resulting systemic sIgA is distributed via common mucosal immune pathways to the respiratory and nasopharyngeal mucosa, where it neutralizes ascending pathogens before middle ear invasion occurs. Importantly, the use of an inactivated preparation eliminates the theoretical risk of bacterial translocation, conferring an additional safety advantage in immunologically immature infants.

In contrast, SS-K12 operates through a fundamentally distinct *in situ* defense strategy. As one of the earliest commensal colonizers of the human oropharynx, SS-K12 produces two high-molecular-weight bacteriocin-like inhibitory substances (BLIS)—salivaricin A2 and salivaricin B—that directly suppress the growth of key AOM pathogens including *S. pneumoniae, S. pyogenes*, and *H. influenzae*, with *in vitro* inhibitory activity demonstrated against approximately 48% of clinical otitis media isolates (11). Beyond direct antimicrobial activity, SS-K12 establishes competitive exclusion at the nasopharyngeal epithelium by occupying receptor sites and depleting nutritional niches, while simultaneously modulating local inflammatory responses through upregulation of IFN-γ and suppression of IL-8 (40). Clinical data indicate that 90-day SS-K12 supplementation reduces streptococcal pharyngotonsillitis incidence by 65–90% over the intervention and subsequent follow-up period (41).

The stark underperformance of BB-12 in the primary outcome, where it failed to demonstrate any statistically significant benefit (OR = 1.05, SUCRA = 23.7%), provides a compelling counterpoint. BB-12 colonizes primarily the lower gastrointestinal tract and lacks the specific surface adhesins required for stable nasopharyngeal attachment (42). Its systemic immunostimulatory capacity appears insufficient to generate protective sIgA concentrations at the distal respiratory mucosa and eustachian tube orifice. This is further corroborated by prior RCT data showing that BB-12-containing combinations failed to reduce nasopharyngeal carriage of *S. pneumoniae* or *H. influenzae* and were associated with a paradoxical increase in *M. catarrhalis* carriage (43). These observations compellingly illustrate that colonization site specificity and the presence of targeted antimicrobial effectors are indispensable determinants of AOM prevention efficacy.

Our findings contextualize and resolve the long-standing discrepancies in the prior literature. The 2019 Cochrane review by Scott et al. (3) reported an overall risk reduction of 23% (RR 0.77, 95% CI: 0.63–0.93) across 17 RCTs, yet with I^2^ = 72%. Mosquera et al. (9) similarly observed approximately 20% risk reduction across 16 RCTs but reported I^2^ = 100% and found no significant benefit for antibiotic use or other secondary endpoints. Both analyses employed a class-level pooling strategy that combined mechanistically heterogeneous strains into a single intervention node—inevitably diluting signal from high-efficacy strains (CBA-L74, SS-K12) with noise from ineffective preparations. The present NMA, by disaggregating the evidence to the strain level, resolves this heterogeneity and identifies precisely which interventions drive, and which suppress, the aggregate pooled signal.

### Antibiotic prescription rates

The finding that Multi-strain combinations ranked first (SUCRA = 87.1%) and LGG second (SUCRA = 65.3%) for antibiotic reduction—both significantly superior to SS-K12 in direct pairwise comparisons—reflects a mechanistic complementarity that diverges from the AOM-specific results. Multi-strain preparations, by combining organisms distributed across distinct gastrointestinal and respiratory ecological niches, establish broader colonization resistance through spatial redundancy and cross-feeding metabolic cascades (44, 45). The presentation of diverse PAMPs to multiple pattern recognition receptors simultaneously activates a more balanced Th1/Th2/Treg immune architecture, suppresses pro-inflammatory cytokine release, and reinforces mucosal barrier integrity at multiple sites (44). This broad immunological remodeling reduces the severity of concurrent viral respiratory infections and attenuates the systemic inflammatory burden that drives empirical antibiotic prescribing—even in the absence of bacteriologically confirmed AOM (44).

LGG's relative advantage in reducing antibiotic use is biologically consistent with its well-characterized SpaCBA pili, which mediate stable intestinal adhesion and sustained secretion of the p40 effector protein in preclinical models (46); the clinical contribution of these mechanisms to the magnitude of effect observed in our network cannot, however, be directly inferred from the present synthesis. p40 transactivates the epidermal growth factor receptor via ADAM17, activating PI3K/Akt anti-apoptotic signaling, thereby sustaining epithelial barrier integrity under inflammatory challenge ^∧^31^∧^. LGG's exopolysaccharides further suppress NF-κB and p38/MAPK pathways, attenuating global inflammatory responses (46). Most relevantly, IgA-committed B lymphocytes activated in the gut-associated lymphoid tissue migrate via mucosal homing receptors to bronchus-associated lymphoid tissue and the nasopharyngeal mucosa, where they generate local sIgA responses that limit viral replication and reduce the progression to secondary bacterial superinfection—the proximate trigger for antibiotic prescribing (47, 48).

The paradoxically poor performance of SS-K12 in the antibiotic outcome reflects the mismatch between its mechanism and the clinical prescribing pathway. SS-K12 effectively suppresses specific bacterial pathogens at the oropharyngeal surface but lacks broad antiviral and systemic anti-inflammatory capacity ^∧^9^∧^. Since the majority of pediatric antibiotic prescriptions for AOM and respiratory illness are driven by systemic viral illness severity—fever, irritability, and persistent otalgia—rather than bacteriological confirmation, an intervention that reduces bacterial colonization without ameliorating the broader inflammatory phenotype will fail to lower the prescribing threshold that clinicians apply (41).

### Exploratory outcomes

The consistent top-ranking of CBA-L74 across both AOM (SUCRA 89.3%) and AGE (SUCRA 95.9%) is biologically coherent. For AGE, CBA-L74 operates in its primary anatomical domain: direct upregulation of colonic tight junction proteins confers structural resistance to enteropathogen-mediated epithelial disruption, while the sustained induction of HBD-2 and LL-37 provides broad-spectrum antimicrobial coverage at the intestinal surface (37, 39). The same sIgA and innate immune amplification cascades that protect the respiratory mucosa simultaneously defend the intestinal mucosa against viral and bacterial enteropathogens, explaining the coherent cross-outcome benefit (37).

LGG's significant reductions in both RTI (OR = 0.56) and AGE (OR = 0.58) reflect its well-characterized multi-target effector repertoire: p75 protein with chitinase activity against fungal cell walls, exopolysaccharide-mediated antioxidant and anti-inflammatory effects, and SpaCBA-dependent mucosal adhesion that enables prolonged immunological priming across gastrointestinal and respiratory compartments (46, 49). The convergence of CBA-L74 and LGG as top performers across multiple infection categories reinforces the concept that durable, mechanism-grounded mucosal immune education—whether through postbiotic stimulation or live colonization—confers benefits that transcend single-disease specificity.

## Limitations

Several limitations merit consideration. First, the star-shaped network topology—with all active nodes connected exclusively through Placebo—precluded closed-loop consistency checks and rendered all between-strain comparisons dependent solely on indirect evidence. The validity of these indirect comparisons rests on the transitivity assumption that the distribution of effect modifiers—participant age, day-care vs. community setting, baseline AOM risk, CFU dose, formulation vehicle, and administration route—is broadly comparable across studies; while no overt violations were detected on qualitative inspection (Supplementary Appendix 7), residual intransitivity cannot be ruled out, and all between-strain estimates and SUCRA-based rankings should therefore be interpreted as hypothesis-generating rather than confirmatory. This caveat is particularly germane to nodes informed by a single trial (SS-24SMB) or by trials at high risk of bias (SS-K12), in which SUCRA probabilities can shift substantially with the addition or exclusion of a single study, as observed in our sensitivity analysis. Second, the number of contributing RCTs was markedly unbalanced across nodes; SS-24SMB was supported by only a single trial, substantially limiting the reliability of its effect estimate and SUCRA ranking. Third, two studies rated as high overall risk of bias [Di Pierro et al. (31); Karpova et al. (33)] contributed to the SS-K12 node, potentially inflating its apparent efficacy for the primary AOM outcome. Fourth, CBA-L74 is a heat-inactivated postbiotic that does not satisfy the conventional WHO/FAO definition of a live probiotic, introducing a conceptual boundary issue when interpreted within a probiotic-focused comparative framework. Fifth, substantial clinical heterogeneity across included trials limits the direct extrapolation of ranked estimates to specific clinical protocols. Daily CFU dose varied by more than two orders of magnitude (1–2 × 10^8^ to 4.1 × 10^10^), formulations spanned fermented milk, fermented rice, chewable tablets, capsules, slow-release lozenges, infant formula, and nasal sprays, and administration routes encompassed oral, oromucosal, and intranasal delivery—each of which is mechanistically distinct (e.g., intranasal SS-24SMB and α-Strep target nasopharyngeal colonization, whereas oral CBA-L74 acts via the gut–lung immune axis). Intervention durations ranged from 30 days to 23 months, and follow-up from 3 to 21 months. These differences plausibly contribute to the moderate between-study heterogeneity observed within several nodes and are an additional reason to interpret single-trial nodes and SUCRA-based rankings with caution. Finally, outcome ascertainment heterogeneity is particularly consequential for low-frequency endpoints such as tympanostomy tube placement, where event counts in several trials were too low for any single study to inform the network meaningfully.

### Methodological innovation and clinical implications

To our knowledge, this is the first NMA in the international literature to evaluate probiotic and postbiotic interventions for pediatric AOM prevention at the individual strain level. Prior strain-level NMAs in adjacent fields—including irritable bowel syndrome (50, 51) and Helicobacter pylori eradication (52)—have demonstrated the transformative value of this approach in revealing intra-class heterogeneity concealed by conventional meta-analyses. By applying this framework to AOM, we resolve the extreme statistical heterogeneity that has rendered prior class-level pooled estimates clinically uninterpretable.

The SUCRA and *P*-score rankings generated by this analysis translate complex probabilistic comparisons into an actionable efficacy hierarchy. Based on these findings, we propose the following strain-specific clinical guidance. For the primary goal of reducing AOM episode incidence, CBA-L74 or SS-K12 should be prioritized; CBA-L74's inactivated formulation confers additional safety advantages in very young or immunocompromised infants (36). For the pragmatic goal of reducing antibiotic prescriptions and preventing chronic disease progression, Multi-strain combinations or LGG are the interventions of choice. For simultaneous prevention of AOM and AGE in high-risk infants in congregate care settings, CBA-L74 offers the broadest cross-outcome protection supported by the present evidence base.

Future research should prioritize direct head-to-head RCTs comparing the leading candidates, particularly a mechanistically rational combination of CBA-L74 (systemic immunological priming via the gut–lung axis) with SS-K12 (local oropharyngeal bacteriocin-mediated defense)—a dual-axis strategy that may provide synergistic protection against the full AOM pathogenic cascade. Mandatory standardization of strain-level reporting in all future publications, including precise strain designations, CFU dosing, formulation matrices, and duration of follow-up, is essential to enable valid evidence synthesis and to prevent the perpetuation of the reporting ambiguity that has confounded the field to date.

## Conclusion

This first strain-level NMA of probiotic interventions for pediatric AOM prevention demonstrates that efficacy is neither uniform nor a class-wide effect. CBA-L74, a heat-inactivated postbiotic, emerged as the most consistently effective and safest intervention for reducing AOM incidence and AGE across all analyses, operating through gut–lung axis immunomodulation that does not require viable bacterial colonization. Multi-strain combinations and LGG were the superior choices for reducing antibiotic prescriptions, reflecting their broader systemic anti-inflammatory and mucosal IgA-stimulating capacity. SS-K12 showed significant AOM prevention in the primary analysis, but its effect was substantially attenuated after exclusion of high risk-of-bias trials, warranting caution. BB-12 demonstrated no meaningful benefit across any outcome, underscoring the critical role of colonization site specificity in determining upper respiratory protection. These findings, although derived predominantly from indirect comparisons within a star-shaped network and supported by low-to-moderate certainty under CINeMA, challenge the prevailing class-level approach to probiotic prescription and provide a preliminary, hypothesis-generating framework for strain-specific clinical decision-making that should be confirmed by future head-to-head trials before being adopted into definitive practice guidance. Future research should prioritize direct head-to-head RCTs, standardized strain-level reporting, and exploration of dual-axis strategies combining gut-mediated systemic immunopriming with local oropharyngeal bacteriocin defense.

## Data Availability

The original contributions presented in the study are included in the article/Supplementary material, further inquiries can be directed to the corresponding author.
