# Supplementary material for: Comparative efficacy of different probiotic strains and preparations for prevention of acute otitis media in children: a systematic review and network meta-analysis
Source: Front Nutr. 2026 Jul 3;13:1847652. doi: 10.3389/fnut.2026.1847652 (PMC13375890; doi:10.3389/fnut.2026.1847652)
Supplement: Supplementary file 1 [file Table_1.docx]

**Appendix 1: Search strategy**

**Table S1. Search strategy of PubMed**

| **#** | **Searches** |
| --- | --- |
| 1 | ("Probiotics"[Mesh] OR probiotic*[tiab] OR "Lactobacillus"[Mesh] OR (lactobacil*[tiab] OR rhamnosus[tiab] OR paracasei[tiab] OR reuteri[tiab] OR acidophilus[tiab] OR salivarius[tiab] OR fermentum[tiab] OR plantarum[tiab] OR helveticus[tiab] OR casei[tiab]) OR "Bifidobacterium"[Mesh] OR (bifidobacter*[tiab] OR bifidus[tiab]) OR "Streptococcus"[Mesh] OR (streptococcus[tiab] OR salivarius[tiab]) OR "Bacillus"[Mesh] OR bacillus[tiab] OR "Saccharomyces"[Mesh] OR saccharomyces[tiab] OR "Enterococcus"[Mesh] OR enterococcus[tiab] OR (LGG[tiab] OR "BB-12"[tiab] OR "K12"[tiab] OR "24SMB"[tiab] OR "CBA L74"[tiab] OR "DSM 17938"[tiab] OR "ATCC 55730"[tiab] OR "alpha-streptococci"[tiab] OR "alpha haemolytic streptococci"[tiab] OR "interfering bacteria"[tiab] OR bacteriotherapy[tiab] OR "live microorganism*"[tiab])) |
| 2 | ("Otitis Media"[Mesh] OR "Otitis Media with Effusion"[Mesh] OR "Otitis Media, Suppurative"[Mesh] OR (("otitis media"[tiab] OR "middle ear infection*"[tiab] OR "middle ear disease*"[tiab] OR "ear infection*"[tiab]) AND (acute[tiab] OR recurrent[tiab] OR chronic[tiab] OR suppurative[tiab])) OR "AOM"[tiab] OR "rAOM"[tiab] OR "acute otitis media"[tiab] OR "recurrent otitis media"[tiab] OR "tympanostomy"[tiab] OR "myringotomy"[tiab] OR "ear tube*"[tiab] OR "grommet*"[tiab]) |
| 3 | ("Child"[Mesh] OR "Adolescent"[Mesh] OR "Infant"[Mesh] OR "Minors"[Mesh] OR "Pediatrics"[Mesh] OR "Puberty"[Mesh] OR "Schools"[Mesh] OR (baby[tiab] OR babies[tiab] OR child*[tiab] OR pediatric*[tiab] OR paediatric*[tiab] OR peadiatric*[tiab] OR infan*[tiab] OR neonat*[tiab] OR newborn*[tiab] OR kid*[tiab] OR adolescen*[tiab] OR preschool[tiab] OR pre-school[tiab] OR toddler*[tiab] OR boy*[tiab] OR girl*[tiab] OR teen*[tiab] OR minors[tiab] OR prepubescen*[tiab] OR postpubescen*[tiab] OR pubescen*[tiab] OR youth*[tiab] OR young[tiab] OR student*[tiab] OR schoolchild*[tiab] OR "school age"[tiab] OR underage*[tiab] OR "under 18"[tiab] OR "under 16"[tiab])) |
| 4 | ("Randomized Controlled Trial"[Publication Type] OR "Controlled Clinical Trial"[Publication Type] OR (randomized[tiab] OR randomly[tiab] OR placebo[tiab] OR trial[tiab] OR groups[tiab] OR "drug therapy"[sh])) |
| 5 | #1 AND #2 AND #3 AND #4 |

**Appendix 2: Risk of bias of randomized clinical trials**

**Table S2: Study level risk of bias assessment using Cochrane risk of bias tool 2.0 for assessing risk of bias of randomized clinical trials**

| Study ID | Randomization process | Deviations from intended interventions | Missing outcome data | Measurement of the outcome | Selection of the reported result | Overall |
| --- | --- | --- | --- | --- | --- | --- |
| **Hatakka 2001** | Low | Low | Low | Low | Low | Low |
| **Roos 2001** | Low | Low | Some concerns | Low | Low | Some concerns |
| **Tano 2002** | Low | Low | Some concerns | Low | Low | Some concerns |
| **Hatakka 2007** | Low | Low | Low | Low | Low | Low |
| **Stecksén-Blicks 2009** | Low | Low | Some concerns | Low | Low | Some concerns |
| **Rautava 2009** | Low | Some concerns | Some concerns | Low | Low | Some concerns |
| **Hojsak 2010** | Low | Low | Low | Low | Low | Low |
| **Maldonado 2012/2015** | Low | Some concerns | Some concerns | Low | Low | Some concerns |
| **Cohen 2013** | Low | Low | Low | Low | Low | Low |
| **Marchisio 2015** | Low | Some concerns | Low | Low | Low | Some concerns |
| **Nocerino 2015** | Low | Low | Low | Low | Low | Low |
| **Taipale 2011/2015** | Low | High | High | Some concerns | Low | High |
| **Di Pierro 2016** | Some concerns | High | Low | High | Low | High |
| **Hojsak 2015** | Low | Low | Low | Low | Low | Low |
| **Karpova 2015** | Some concerns | High | Some concerns | High | Low | High |
| **Corsello 2017** | Low | Low | Low | Low | Low | Low |
| **Sarlin 2023** | Low | Low | Low | Low | Low | Low |
| **Paduchová 2024** | Low | Some concerns | Some concerns | Low | Some concerns | Some concerns |

**Appendix 3: Evaluation of inconsistency and heterogeneity**

**Table S3.1: Global consistency**

| **Clinical outcome** | **Chi square** | **P value** | **Tau²** |
| --- | --- | --- | --- |
| Incidence of AOM episodes | 0.15 | 0.6961 | 0.37177 |
| Antibiotic prescription rates | 2.54 | 0.111 | ＜0.04 |
| Tympanostomy tube placement rates | 0.05 | 0.8168 | ＜0.04 |
| Incidence of respiratory tract infections (RTI) | 13.43 | 0.2314 | 0.14691 |
| Incidence of gastrointestinal infections / acute gastroenteritis (AGE) | 0.68 | 0.4085 | ＜0.04 |

**Appendix 4: Funnel plots**

**Figure S4.1: Funnel plot of Incidence of AOM episodes**


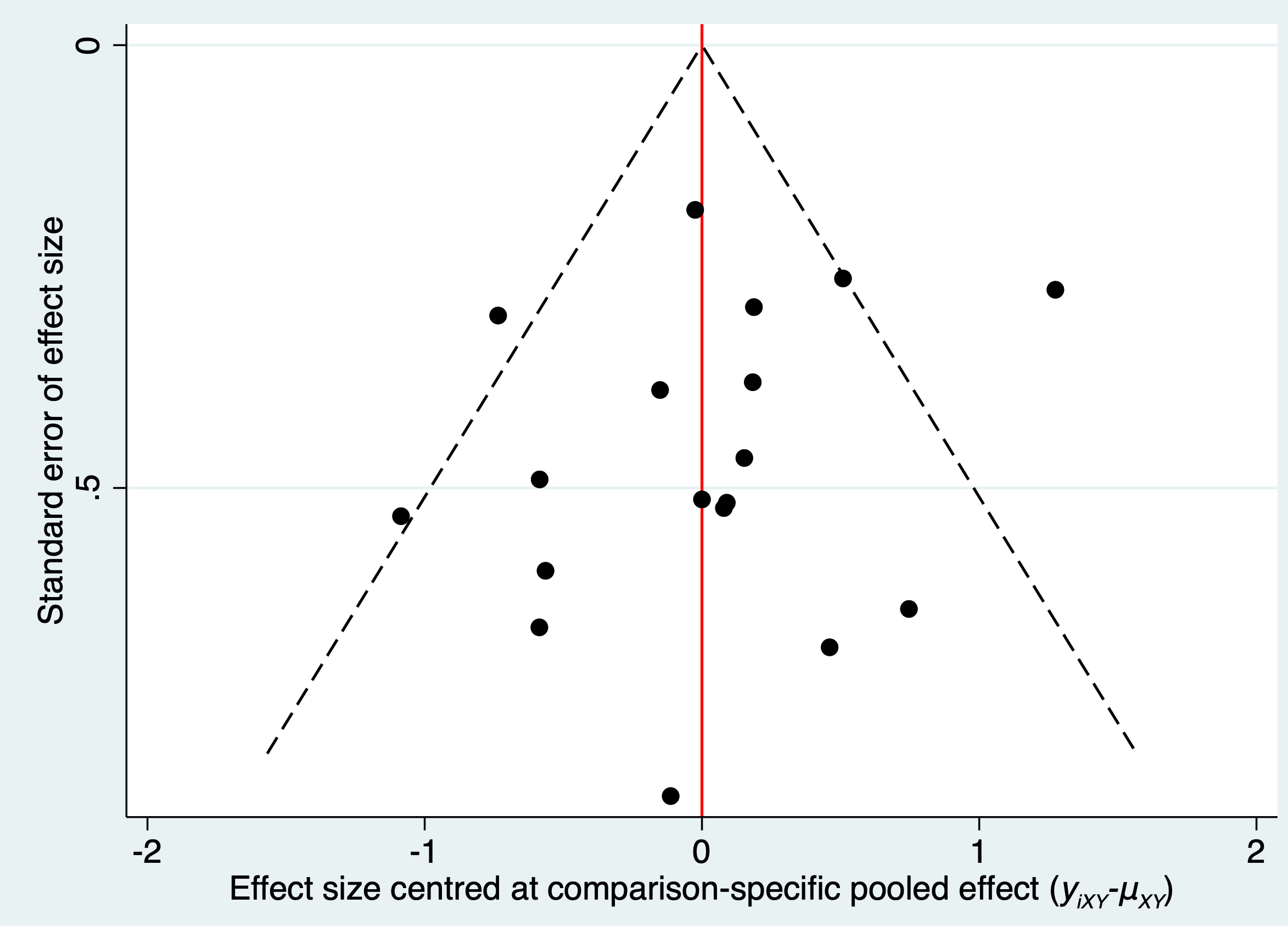


**Figure S4.2: Funnel plot of Antibiotic prescription rates**


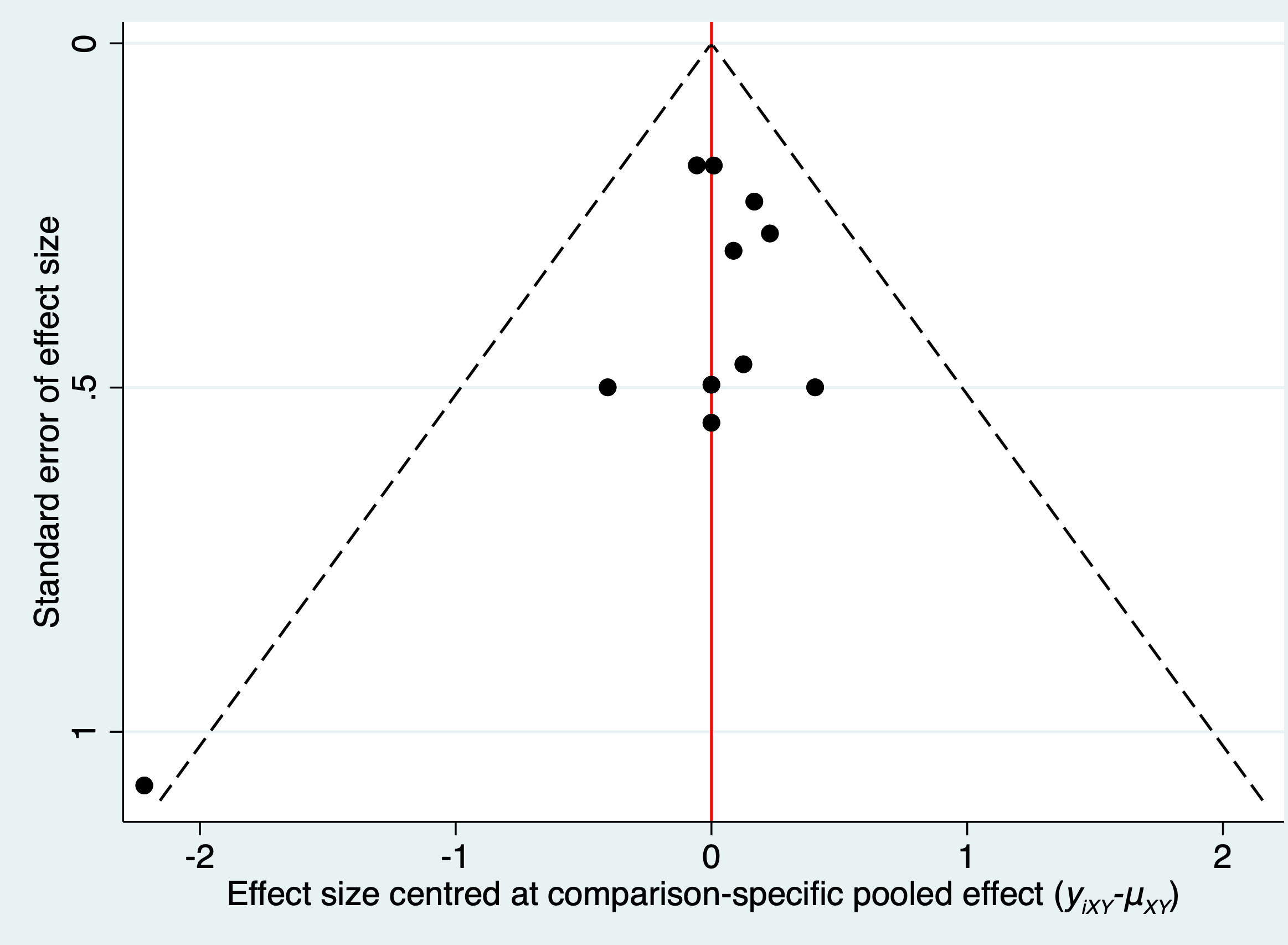


**Figure S4.3: Funnel plot of RTI**


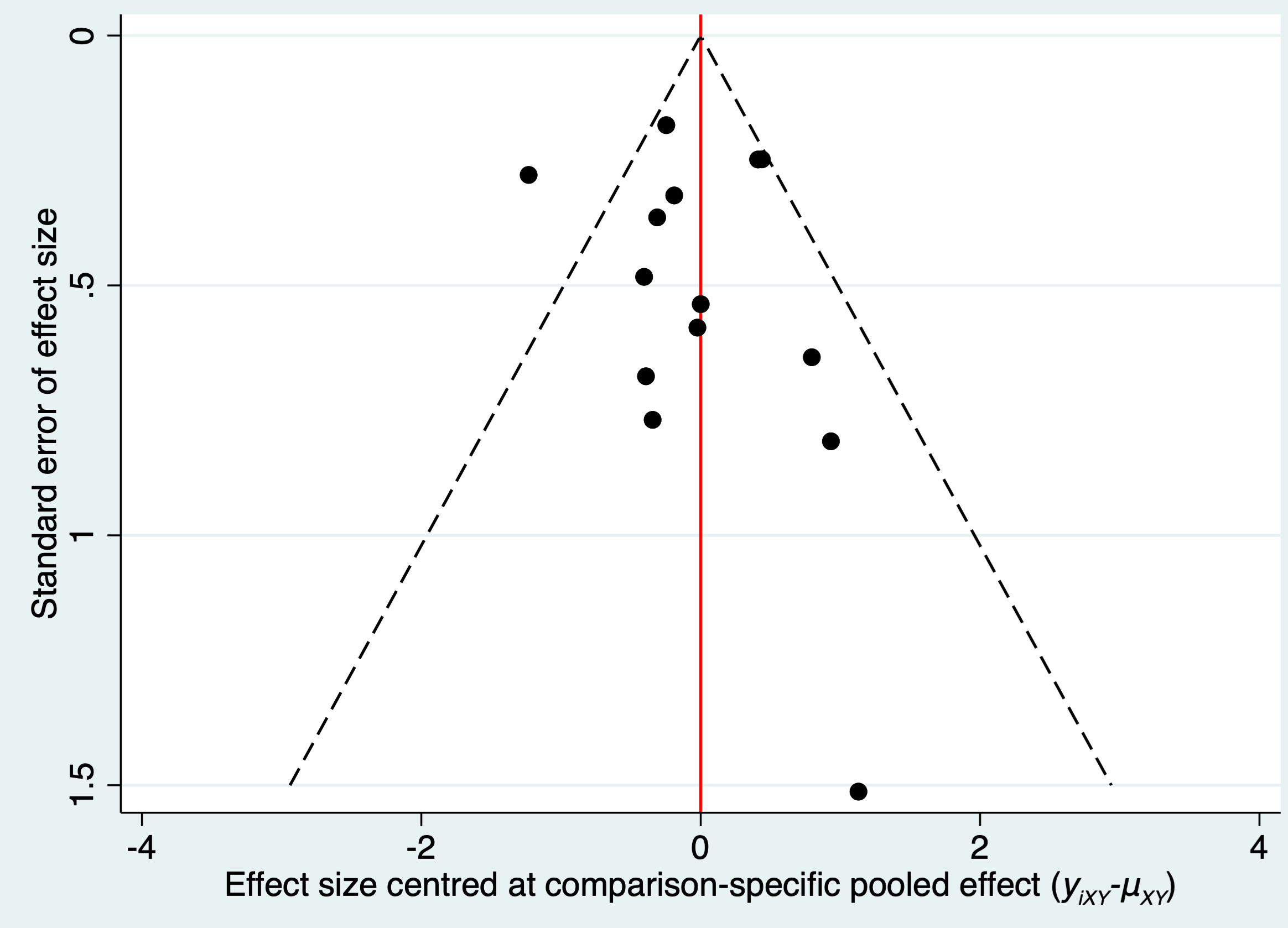


**Figure S4.4: Funnel plot of AGE**


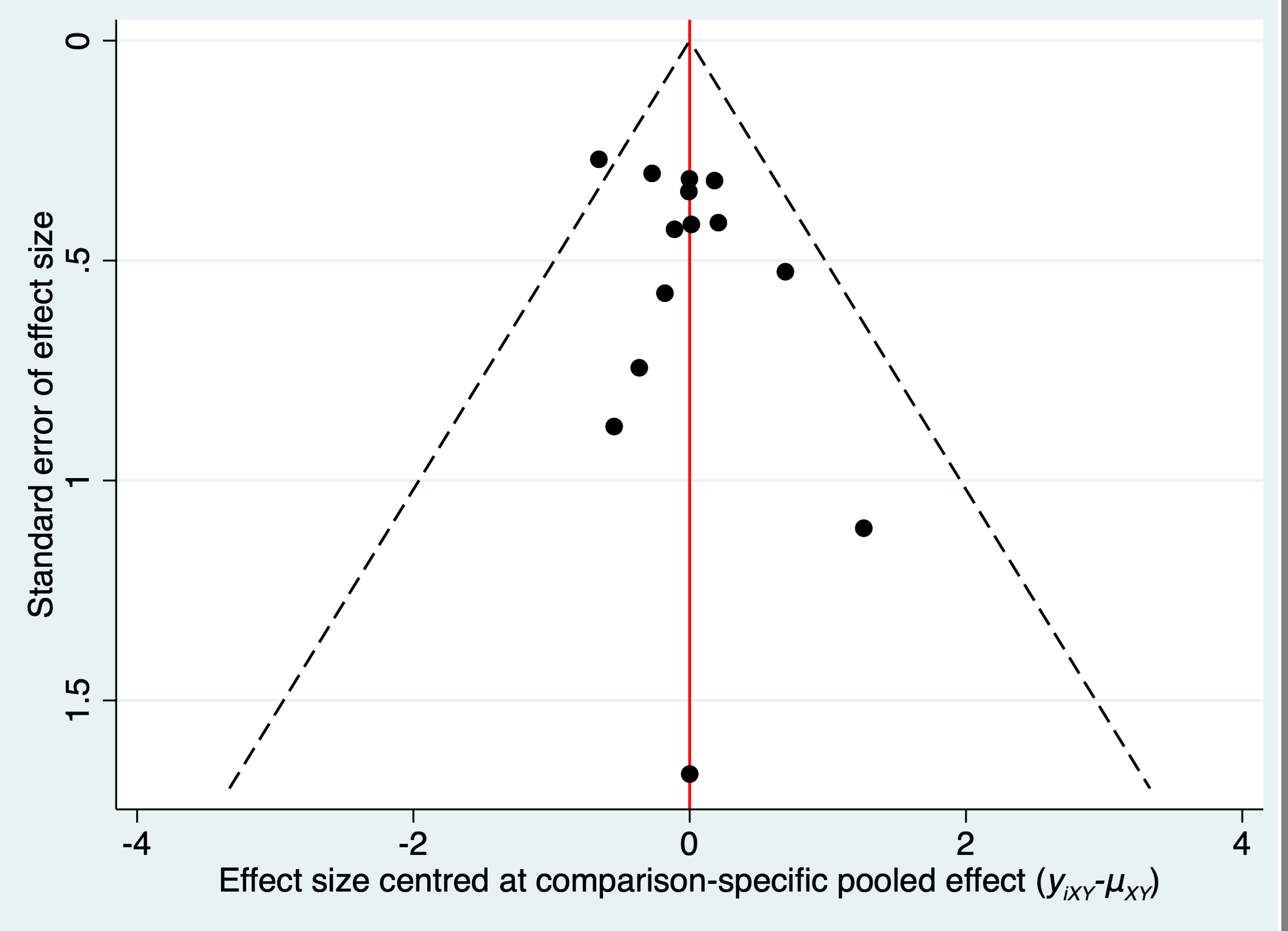


**Appendix 5: SUCRA and cumulative probability plots**

**Figure S5.1: Incidence of AOM episodes**


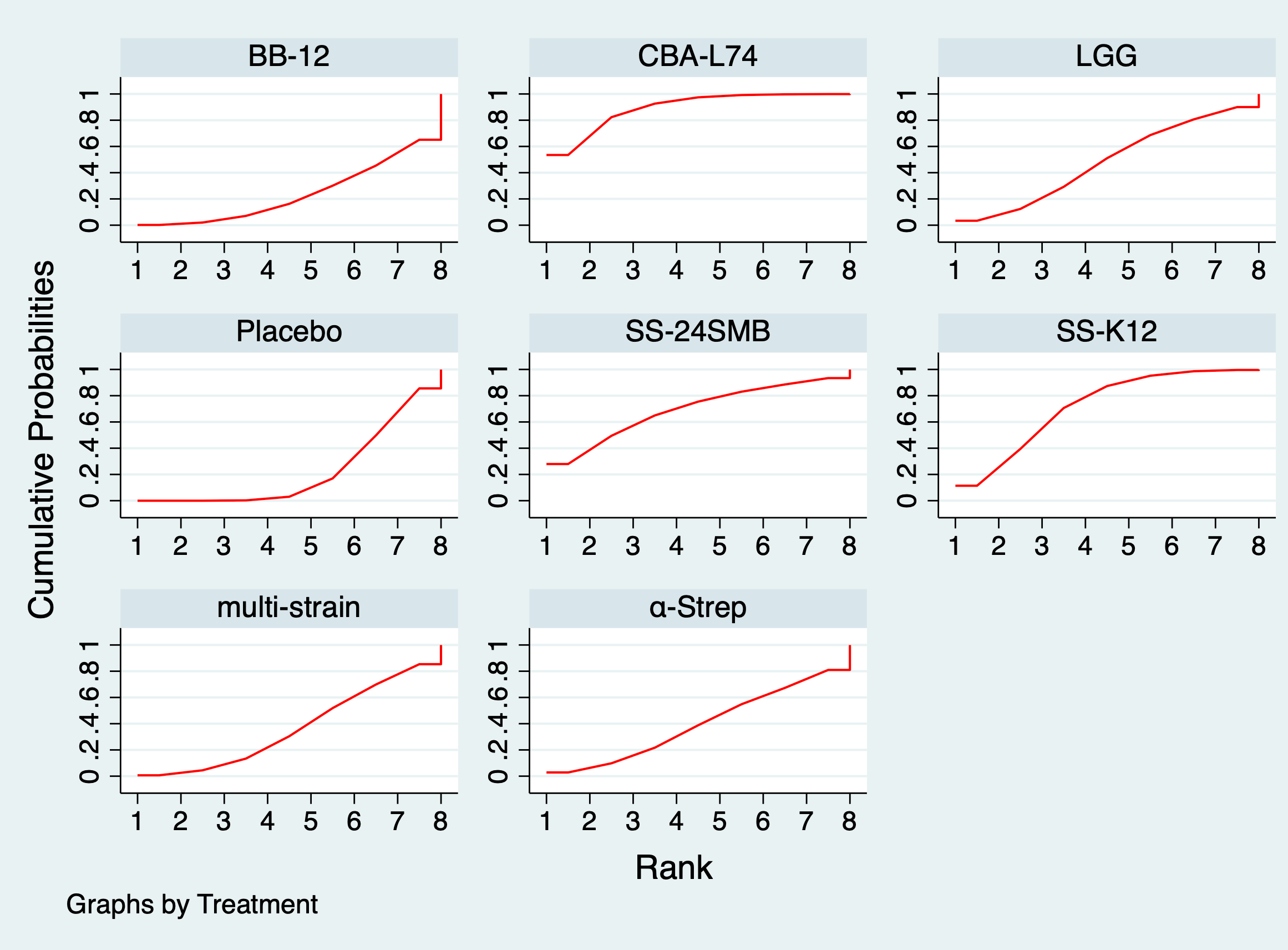


| Treatm~t | SUCRA | PrBest | MeanRank |
| --- | --- | --- | --- |
| BB-12 | 23.7 | 0.2 | 6.3 |
| CBA-L74 | 89.3 | 53.5 | 1.8 |
| LGG | 47.9 | 3.4 | 4.6 |
| Placebo | 22.2 | 0 | 6.4 |
| SS-24SMB | 69 | 27.9 | 3.2 |
| SS-K12 | 71.8 | 11.4 | 3 |
| multi-strain | 36.6 | 0.7 | 5.4 |
| α-Strep | 39.5 | 2.8 | 5.2 |

**Figure S5.2: Antibiotic prescription rates**


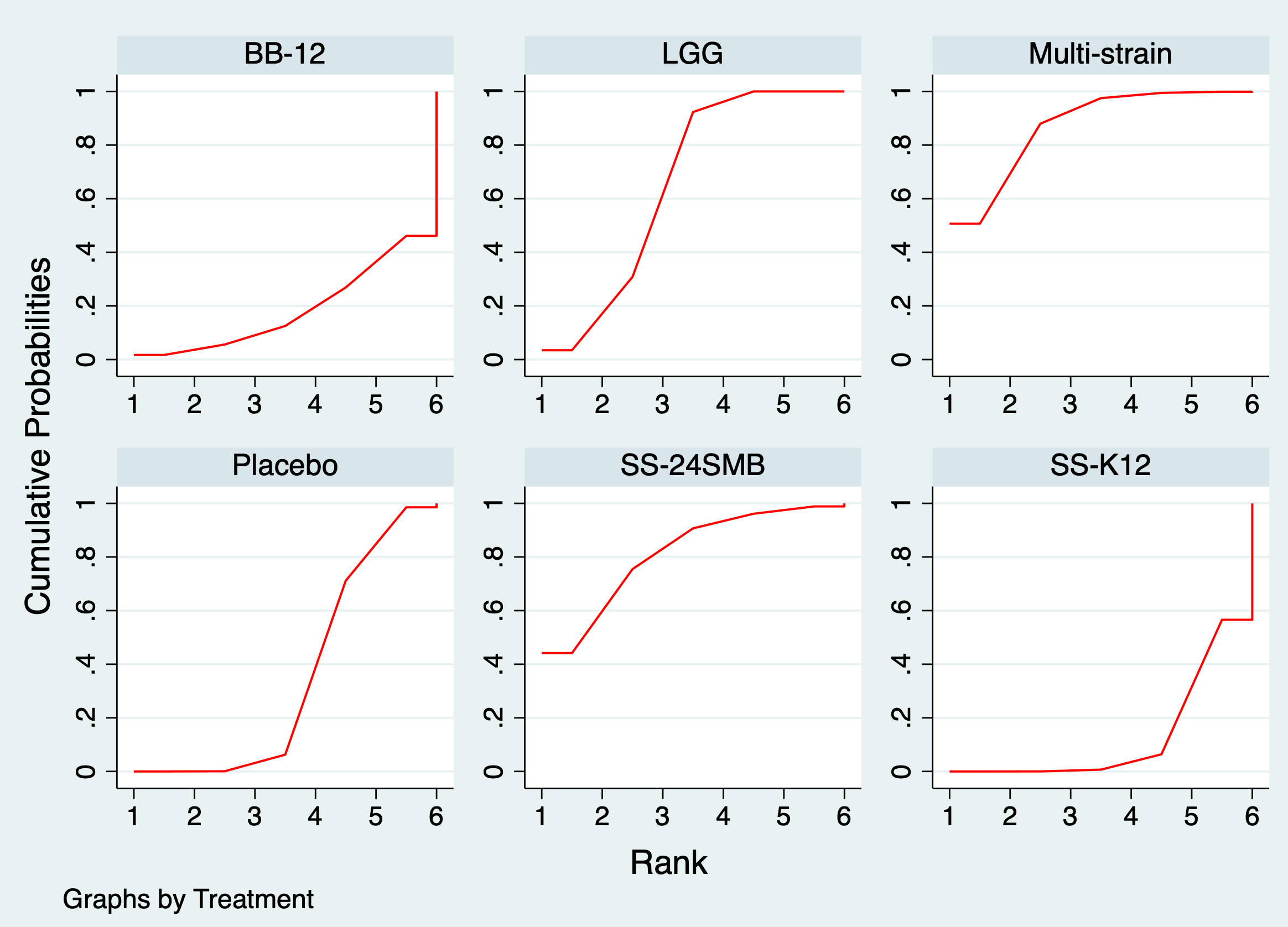


| Treatm~t | SUCRA | PrBest | MeanRank |
| --- | --- | --- | --- |
| BB-12 | 18.6 | 1.7 | 5.1 |
| LGG | 65.3 | 3.5 | 2.7 |
| Multi-strain | 87.1 | 50.7 | 1.6 |
| Placebo | 35.2 | 0 | 4.2 |
| SS-24SMB | 81.1 | 44.1 | 1.9 |
| SS-K12 | 12.7 | 0 | 5.4 |

**Figure S5.3: Tympanostomy tube placement rates**


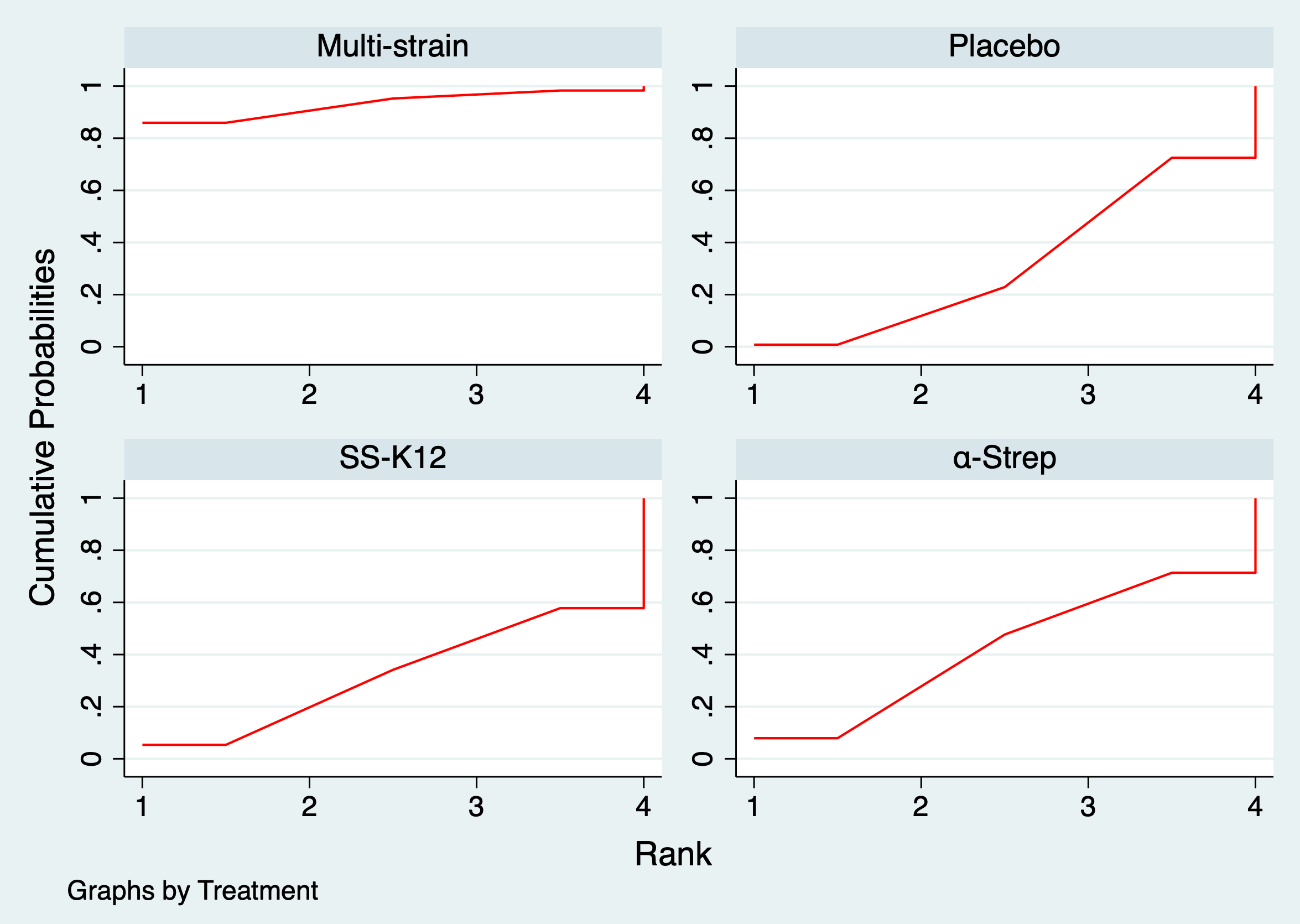


| Treatm~t | SUCRA | PrBest | MeanRank |
| --- | --- | --- | --- |
| Multi-strain | 93.2 | 85.9 | 1.2 |
| Placebo | 32.1 | 0.8 | 3 |
| SS-K12 | 32.4 | 5.4 | 3 |
| α-Strep | 42.3 | 7.9 | 2.7 |

**Figure S5.4: Incidence of respiratory tract infections (RTI)**


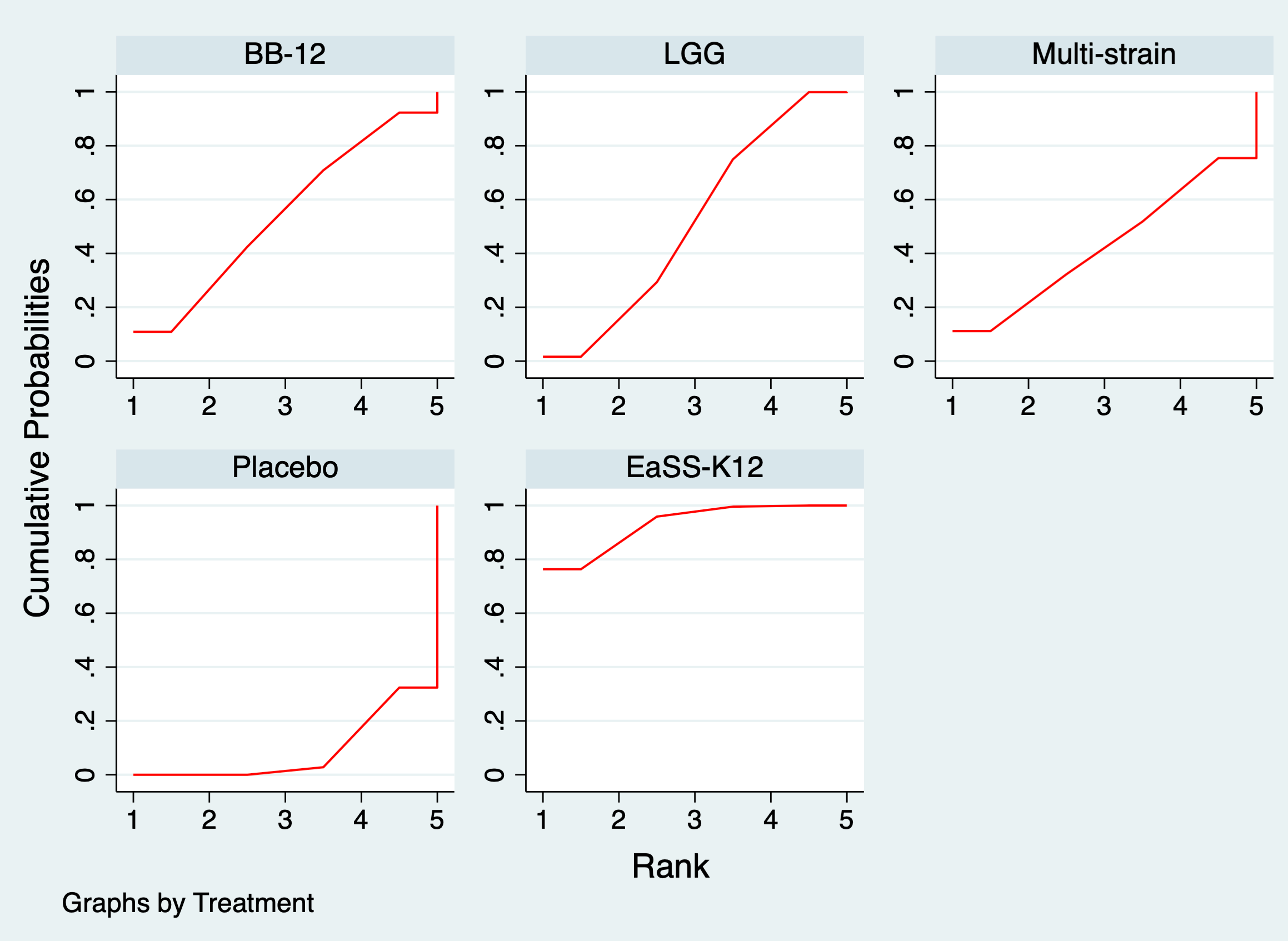


| Treatm~t | SUCRA | PrBest | MeanRank |
| --- | --- | --- | --- |
| BB-12 | 54.1 | 10.9 | 2.8 |
| LGG | 51.5 | 1.6 | 2.9 |
| Multi-strain | 42.7 | 11.1 | 3.3 |
| Placebo | 8.8 | 0 | 4.6 |
| SS-K12 | 93 | 76.3 | 1.3 |

**Figure S5.5: Incidence of gastrointestinal infections / acute gastroenteritis (AGE)**


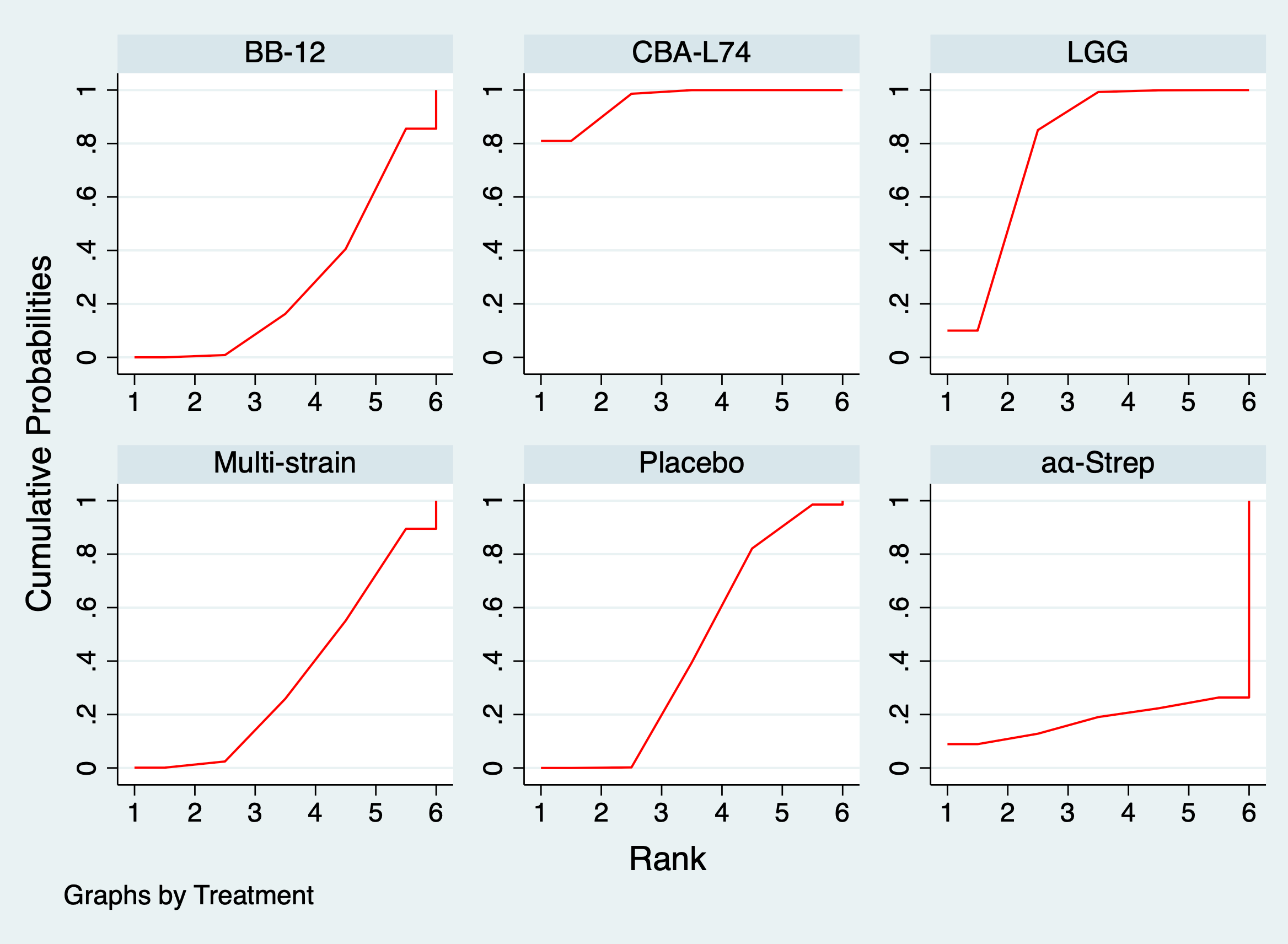


| Treatm~t | SUCRA | PrBest | MeanRank |
| --- | --- | --- | --- |
| BB-12 | 28.6 | 0 | 4.6 |
| CBA-L74 | 95.9 | 81 | 1.2 |
| LGG | 78.8 | 10 | 2.1 |
| Multi-strain | 34.6 | 0.1 | 4.3 |
| Placebo | 44.1 | 0 | 3.8 |
| α-Strep | 17.9 | 8.9 | 5.1 |

**Appendix 6: League tables**

**Table S6.1: League table for Incidence of AOM episodes**

| BB-12 | 0.26 (0.07,0.93) | 0.64 (0.17,2.37) | 0.95 (0.39,2.32) | 0.39 (0.06,2.35) | 0.41 (0.13,1.35) | 0.78 (0.23,2.59) | 0.76 (0.18,3.17) |
| --- | --- | --- | --- | --- | --- | --- | --- |
| **3.89 (1.08,14.04)** | CBA-L74 | 2.49 (0.66,9.48) | 3.70 (1.47,9.37) | 1.51 (0.25,9.30) | 1.61 (0.48,5.37) | 3.02 (0.89,10.31) | 2.97 (0.70,12.65) |
| 1.56 (0.42,5.77) | 0.40 (0.11,1.52) | LGG | 1.49 (0.57,3.89) | 0.61 (0.10,3.80) | 0.65 (0.19,2.21) | 1.21 (0.35,4.25) | 1.19 (0.27,5.18) |
| 1.05 (0.43,2.55) | **0.27 (0.11,0.68)** | 0.67 (0.26,1.76) | Placebo | 0.41 (0.09,1.95) | 0.44 (0.20,0.94) | 0.82 (0.36,1.83) | 0.80 (0.26,2.44) |
| 2.57 (0.43,15.48) | 0.66 (0.11,4.07) | 1.65 (0.26,10.31) | 2.45 (0.51,11.67) | SS-24SMB | 1.07 (0.19,6.08) | 2.00 (0.34,11.59) | 1.96 (0.29,13.35) |
| 2.41 (0.74,7.82) | 0.62 (0.19,2.07) | 1.55 (0.45,5.29) | **2.30 (1.06,4.96)** | 0.94 (0.16,5.35) | SS-K12 | 1.87 (0.62,5.68) | 1.84 (0.47,7.14) |
| 1.29 (0.39,4.27) | 0.33 (0.10,1.13) | 0.82 (0.24,2.89) | 1.23 (0.55,2.75) | 0.50 (0.09,2.90) | 0.53 (0.18,1.62) | multi-strain | 0.98 (0.25,3.89) |
| 1.31 (0.32,5.43) | 0.34 (0.08,1.44) | 0.84 (0.19,3.66) | 1.25 (0.41,3.80) | 0.51 (0.07,3.47) | 0.54 (0.14,2.11) | 1.02 (0.26,4.04) | α-Strep |

**Table S6.2: League table for Antibiotic prescription rates**

| BB-12 | 0.49 (0.16,1.47) | 0.32 (0.09,1.17) | 0.71 (0.24,2.09) | 0.34 (0.08,1.46) | 0.94 (0.30,2.91) |
| --- | --- | --- | --- | --- | --- |
| 2.05 (0.68,6.17) | LGG | 0.66 (0.32,1.37) | 1.46 (1.17,1.82) | 0.70 (0.26,1.89) | 1.92 (1.28,2.89) |
| 3.09 (0.86,11.16) | 1.51 (0.73,3.12) | Multi-strain | 2.20 (1.10,4.40) | 1.05 (0.32,3.47) | 2.90 (1.34,6.29) |
| 1.41 (0.48,4.14) | **0.69 (0.55,0.86)** | **0.45 (0.23,0.91)** | Placebo | 0.48 (0.18,1.26) | 1.32 (0.94,1.86) |
| 2.94 (0.69,12.56) | 1.43 (0.53,3.89) | 0.95 (0.29,3.13) | 2.09 (0.79,5.52) | SS-24SMB | 2.76 (0.98,7.73) |
| 1.07 (0.34,3.31) | **0.52 (0.35,0.78)** | **0.34 (0.16,0.75)** | 0.76 (0.54,1.07) | 0.36 (0.13,1.02) | SS-K12 |

**Table S6.3: League table for Tympanostomy tube placement rates**

| Multi-strain | 5.12 (0.87,30.05) | 5.17 (0.54,49.32) | 4.38 (0.49,39.53) |
| --- | --- | --- | --- |
| 0.20 (0.03,1.15) | Placebo | 1.01 (0.25,4.09) | 0.86 (0.23,3.16) |
| 0.19 (0.02,1.84) | 0.99 (0.24,3.99) | SS-K12 | 0.85 (0.13,5.73) |
| 0.23 (0.03,2.06) | 1.17 (0.32,4.30) | 1.18 (0.17,7.97) | α-Strep |

**Table S6.4: League table for RTI**

| BB-12 | 1.06 (0.37,2.99) | 1.20 (0.25,5.88) | 1.88 (0.75,4.72) | 0.50 (0.15,1.66) |
| --- | --- | --- | --- | --- |
| 0.95 (0.33,2.67) | LGG | 1.14 (0.29,4.41) | 1.78 (1.19,2.66) | 0.47 (0.21,1.06) |
| 0.83 (0.17,4.07) | 0.88 (0.23,3.41) | Multi-strain | 1.57 (0.43,5.71) | 0.42 (0.10,1.82) |
| 0.53 (0.21,1.33) | **0.56 (0.38,0.84)** | 0.64 (0.18,2.33) | Placebo | 0.27 (0.13,0.54) |
| 1.99 (0.60,6.60) | 2.11 (0.94,4.71) | 2.39 (0.55,10.45) | **3.75 (1.85,7.60)** | SS-K12 |

**Table S6.5: League table for AGE**

| BB-12 | 0.34 (0.18,0.64) | 0.48 (0.25,0.91) | 0.93 (0.45,1.90) | 0.83 (0.50,1.37) | 3.30 (0.12,89.94) |
| --- | --- | --- | --- | --- | --- |
| **2.93 (1.56,5.51)** | CBA-L74 | 1.41 (0.81,2.43) | 2.72 (1.44,5.16) | 2.44 (1.67,3.56) | 9.67 (0.36,259.53) |
| **2.09 (1.10,3.96)** | 0.71 (0.41,1.23) | LGG | 1.94 (1.01,3.71) | 1.73 (1.17,2.58) | 6.88 (0.26,185.00) |
| 1.08 (0.53,2.21) | **0.37 (0.19,0.70)** | **0.52 (0.27,0.99)** | Multi-strain | 0.90 (0.54,1.50) | 3.55 (0.13,97.08) |
| 1.20 (0.73,1.99) | **0.41 (0.28,0.60)** | **0.58 (0.39,0.86)** | 1.12 (0.67,1.87) | Placebo | 3.97 (0.15,104.18) |
| 0.30 (0.01,8.28) | 0.10 (0.00,2.78) | 0.15 (0.01,3.91) | 0.28 (0.01,7.69) | 0.25 (0.01,6.62) | α-Strep |

**Appendix 7: Transitivity assessment of effect modifiers**

Effect modifiers pre-specified in the protocol (participant age, clinical setting, baseline AOM risk, daily CFU dose, formulation vehicle, and administration route) were tabulated for each included RCT and grouped by NMA intervention node (Table S7.1). On qualitative inspection no effect modifier was systematically clustered with a single intervention node in a way that would constitute an overt violation of transitivity. Three patterns merit transparent acknowledgement and are discussed in the Limitations: (i) intranasal delivery was confined to the SS-24SMB and α-Strep nodes; (ii) all otitis-prone / high-risk cohorts were concentrated in the SS-24SMB, α-Strep, Multi-strain (Hatakka 2007), SS-K12 (Karpova 2015) and Multi-strain-formula (Cohen 2013) nodes, whereas the CBA-L74, BB-12 and LGG nodes were informed exclusively by general-paediatric day-care cohorts; (iii) daily CFU dose spanned more than two orders of magnitude (1×10⁸ to ≈4×10¹⁰) across nodes. Residual intransitivity associated with these clusterings cannot be ruled out, and between-strain estimates should be interpreted as hypothesis-generating accordingly.

**Table S7.1: Distribution of effect modifiers across the 18 included RCTs, grouped by NMA intervention node (transitivity assessment).**

| **Study** | **Node** | **Country** | **n** | **Age** | **Clinical setting** | **Baseline AOM risk** | **Daily CFU dose** | **Formulation & route** |
| --- | --- | --- | --- | --- | --- | --- | --- | --- |
| Sarlin 2023 | SS-K12 | Finland | 827 | 1–6 y (mean 4.1) | Day-care centre | General paediatric | 1×10⁹ | Powder/chewable tablet, oral |
| Di Pierro 2016 | SS-K12 | Italy | 222 | 33–45 mo | Kindergarten | General paediatric | ≥1×10⁹ | Slow-release lozenge, oral |
| Karpova 2015 | SS-K12 | Russia | 219 | 6–7 y | Child collective (kindergarten/day-care) | Otitis-prone / high-risk (chronic adenoiditis) | NR (1 tablet/day) | Tablet, oral |
| Marchisio 2015 | SS-24SMB | Italy | 97 | 1–5 y (mean 2.7/3.1) | Clinic / hospital | Otitis-prone | 2×10¹⁰ | Nasal spray, intranasal |
| Hojsak 2015 | BB-12 | Croatia | 210 | 1.4–7.5 y (median 4.6) | Day-care centre | General paediatric | 1×10⁹ | Powder, oral |
| Taipale 2011/2015 | BB-12 | Finland | 109 | 1 mo – 2 y | Community well-baby clinic | General paediatric | 1×10¹⁰ | Tablet via pacifier/spoon, oral |
| Corsello 2017 | CBA-L74 | Italy | 146 | 12–48 mo (mean 33) | Day-care / kindergarten | General paediatric | NR (7 g/day inactivated) | Fermented skim-milk powder, oral |
| Nocerino 2015 | CBA-L74 | Italy | 432 | 12–48 mo (mean 32) | Day-care / kindergarten | General paediatric | ≈4.1×10¹⁰ | Fermented milk / fermented rice powder, oral |
| Hatakka 2001 | LGG | Finland | 571 | 1–6 y (mean 4.5) | Day-care centre | General paediatric | 1–2×10⁸ | Probiotic milk, oral |
| Stecksén-Blicks 2009 | LGG (LB21) | Sweden | 248 | 1–5 y (mean 42 mo) | Day-care centre | General paediatric | 1.5×10⁹ | Milk + capsule supplement, oral |
| Hojsak 2010 | LGG | Croatia | 281 | 13–86 mo (mean 52) | Day-care centre | General paediatric | 1×10⁹ | Fermented milk, oral |
| Cohen 2013 | Multi-strain | France | 224 | 7–13 mo (mean 10) | Outpatient clinic | High-risk infants | ≈10⁷ CFU/g formula | Follow-on formula, oral |
| Rautava 2009 | Multi-strain | Finland | 81 | <2 mo – 12 mo | Community well-baby clinic | General paediatric | 2×10¹⁰ | Capsule (mixed into formula), oral |
| Hatakka 2007 | Multi-strain | Finland | 309 | 10 mo – 6 y (mean 2.4) | Primary care + day-care | Otitis-prone | ≈8–9×10⁹ per strain | Capsule, oral |
| Roos 2001 | α-Strep | Sweden | 130 | 6 mo – 6 y | ENT clinic | Otitis-prone (recurrent AOM) | >5×10⁶ CFU/mL spray | Nasal spray, intranasal |
| Tano 2002 | α-Strep | Sweden | 43 | ≤3 y (mean 21 mo) | ENT clinic | Otitis-prone (recurrent AOM) | >10⁶ CFU/spray | Nasal spray, intranasal |
| Maldonado 2012/2015 | L. fermentum CECT5716 (single-strain — secondary outcomes only) | Spain | 215 | 6–12 mo | Hospital / primary care | General paediatric | 2×10⁸ | Follow-on formula, oral |
| Paduchová 2024 | Multi-strain (secondary outcomes only) | Slovakia | 127 | 3–10 y (mean 4.8) | Kindergarten / day-care | General paediatric | 1.25×10¹⁰ | Chewable tablet, oral |

Appendix 8 sensitive analysis


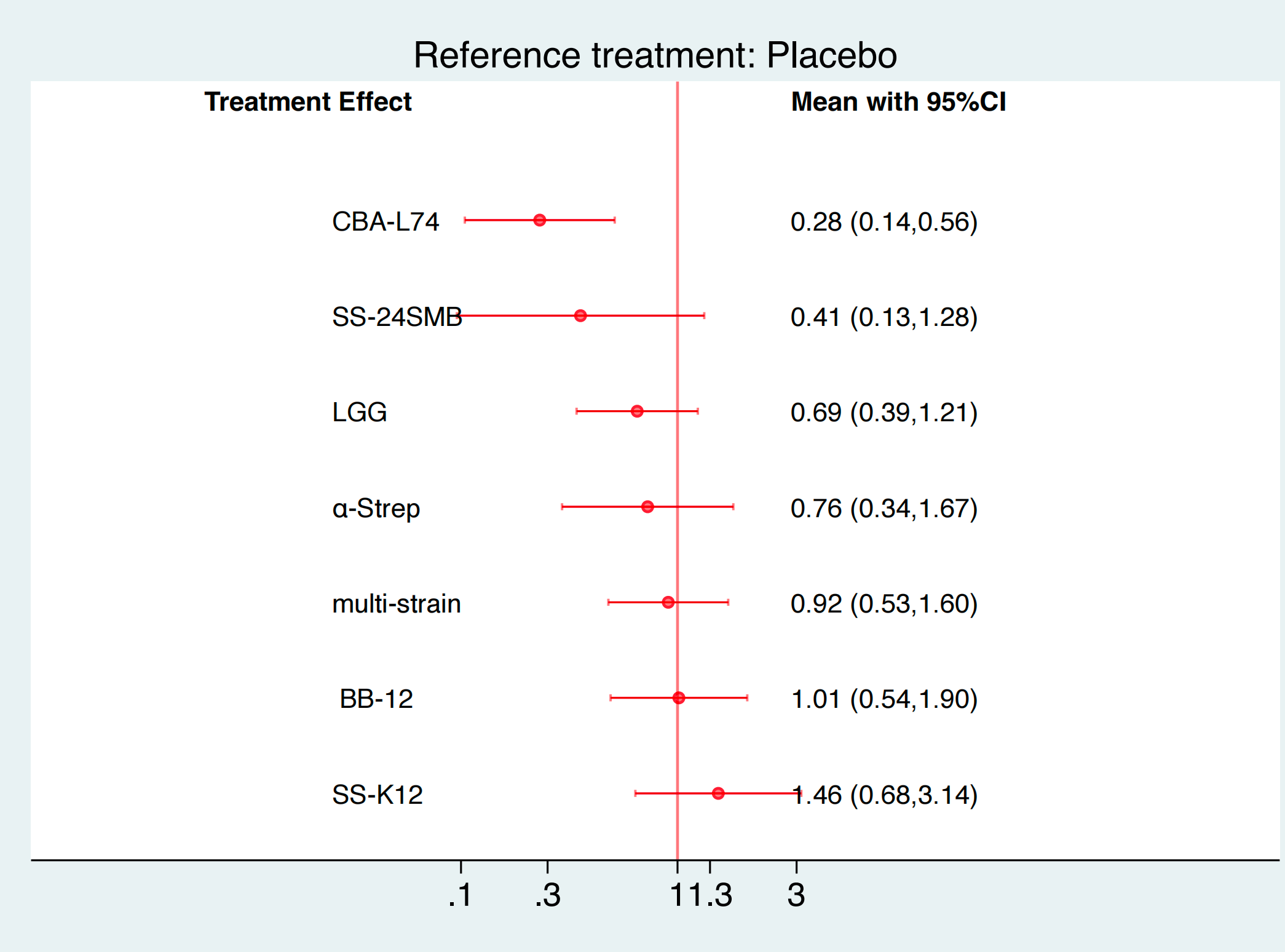


**Appendix 9: Certainty of evidence under the CINeMA framework**

Confidence in the network estimates was assessed using the CINeMA framework across six domains: within-study bias, reporting bias, indirectness, imprecision, heterogeneity, and inconsistency. The certainty rating for each placebo-controlled estimate is summarised below; full domain-level judgements are tabulated in Table S9.1.

Primary outcome (AOM incidence):
 • CBA-L74 vs Placebo — moderate certainty (downgraded once for imprecision).
 • SS-K12 vs Placebo — low certainty (downgraded for within-study bias and imprecision; further attenuated to very low after sensitivity analysis).
 • SS-24SMB vs Placebo — very low certainty (downgraded for imprecision and very sparse direct evidence; single-trial node).
 • α-Strep vs Placebo — very low certainty (downgraded for imprecision and within-study bias).
 • BB-12, LGG, Multi-strain vs Placebo — low certainty (downgraded for imprecision and indirectness).

Antibiotic prescription rates:
 • LGG vs Placebo — moderate certainty (downgraded for imprecision).
 • Multi-strain vs Placebo — low certainty (downgraded for imprecision and heterogeneity in antibiotic-indication definitions).
 • SS-K12, SS-24SMB, BB-12 vs Placebo — low to very low.

Tympanostomy tube placement rates: very low certainty across all contrasts owing to imprecision and few events.

All between-strain (active vs. active) contrasts carried at least one downgrade for indirectness because they relied exclusively on indirect evidence within the star-shaped network.

Table S9.1: Domain-level CINeMA judgements for all network estimates (within-study bias, reporting bias, indirectness, imprecision, heterogeneity, inconsistency).

| **Outcome** | **Contrast** | **Within-study bias** | **Reporting bias** | **Indirectness** | **Imprecision** | **Heterogeneity** | **Inconsistency** | **Overall certainty** |
| --- | --- | --- | --- | --- | --- | --- | --- | --- |
| ***AOM incidence*** | CBA-L74 vs Placebo | No concerns | No concerns | No concerns | Some concerns | No concerns | NA | **Moderate** |
|  | SS-K12 vs Placebo | Major concerns | No concerns | No concerns | Some concerns | Some concerns | NA | **Low** |
|  | BB-12 vs Placebo | Some concerns | No concerns | No concerns | Some concerns | No concerns | NA | **Low** |
|  | LGG vs Placebo | Some concerns | No concerns | No concerns | Some concerns | Some concerns | NA | **Low** |
|  | Multi-strain vs Placebo | Some concerns | No concerns | Some concerns | Some concerns | Some concerns | NA | **Low** |
|  | SS-24SMB vs Placebo | Some concerns | Some concerns | No concerns | Major concerns | NA | NA | **Very low** |
|  | α-Strep vs Placebo | Some concerns | Some concerns | No concerns | Major concerns | No concerns | NA | **Very low** |
| ***Antibiotic prescription*** | LGG vs Placebo | No concerns | No concerns | No concerns | Some concerns | No concerns | NA | **Moderate** |
|  | Multi-strain vs Placebo | Some concerns | No concerns | Some concerns | Some concerns | Some concerns | NA | **Low** |
|  | BB-12 vs Placebo | Some concerns | No concerns | No concerns | Some concerns | No concerns | NA | **Low** |
|  | SS-K12 vs Placebo | Major concerns | No concerns | No concerns | Some concerns | Some concerns | NA | **Very low** |
|  | SS-24SMB vs Placebo | Some concerns | Some concerns | No concerns | Major concerns | NA | NA | **Very low** |
| ***Tympanostomy tube placement*** | Multi-strain vs Placebo | Some concerns | Some concerns | No concerns | Major concerns | No concerns | NA | **Very low** |
|  | α-Strep vs Placebo | Some concerns | Some concerns | No concerns | Major concerns | No concerns | NA | **Very low** |
|  | SS-K12 vs Placebo | Some concerns | Some concerns | No concerns | Major concerns | No concerns | NA | **Very low** |

*Footnote. Within-study bias downgraded for SS-K12 because two of three trials (Di Pierro 2016; Karpova 2015) were open-label with untreated controls; downgraded for α-Strep, SS-24SMB and Multi-strain because contributing trials raised some concerns under RoB 2. Reporting bias rated as Some concerns for SS-24SMB and α-Strep (single- or two-trial nodes preclude small-study assessment). Imprecision rated as Major concerns where the 95% CI crossed both 0.5 and 2.0 or where the node was informed by ≤2 trials with few events. Heterogeneity marked NA where a node was informed by a single trial. Inconsistency is NA across all contrasts because the network has a star-shaped topology without closed loops, precluding direct-versus-indirect comparison; all between-strain estimates therefore rely on indirect evidence and carry implicit downgrading for indirectness which is reflected in the Overall column.*
